# Gene expression profile analysis of gallic acid-induced cell death process

**DOI:** 10.1038/s41598-021-96174-1

**Published:** 2021-08-18

**Authors:** Ho Man Tang, Peter Chi Keung Cheung

**Affiliations:** School of Life Sciences, EG09, Science Centre East Block, The Chinese University of Hong Kong, Shatin, New Territories, Hong Kong, SAR China

**Keywords:** Cancer, Cell biology

## Abstract

Gallic acid is a natural phenolic compound that displays anti-cancer properties in clinically relevant cell culture and rodent models. To date, the molecular mechanism governing the gallic acid-induced cancer cell death process is largely unclear, thus hindering development of novel therapeutics. Therefore, we performed time-course RNA-sequencing to reveal the gene expression profiles at the early (2nd hour), middle (4th and 6th hour), and late (9th hour) stages of the gallic acid-induced cell death process in HeLa cells. By Gene Ontology (GO) and Kyoto Encyclopedia of Genes and Genomes (KEGG) enrichment analyses, we found significant changes in transcription of the genes in different types of cell death pathways. This involved the ferroptotic cell death pathway at the early stage, apoptotic pathway at the middle stage, and necroptotic pathway at the late stage. Metabolic pathways were identified at all the stages, indicating that this is an active cell death process. Interestingly, the initiation and execution of gallic acid-induced cell death were mediated by multiple biological processes, including iron and amino acid metabolism, and the biosynthesis of glutathione, as targeting on these pathways suppressed cell death. In summary, our work provides a dataset with differentially expressed genes across different stages of cell death process during the gallic acid induction, which is important for further study on the control of this cell death mechanism.

## Introduction

Gallic acid is a phenolic acid commonly found in many dietary substances, such as edible mushrooms, fruits, herbs, nuts, tea leaves, and vegetables^1,2^. This natural bioactive compound displays cytotoxicity to multiple types of human cancer cells, including breast cancer^3^, cervical cancer^4^, colon cancer^5^, liver cancer^6^, lung cancer^7^, skin cancer^8^, leukemia^9^, and lymphoma^10^, in cell culture or small animal xerograph models^11^. Of particular interest, studies in rodents revealed that administration of gallic acid at the dosages with the anti-cancer effects has no observed side effect in vivo, as evidenced by normal body and organ weight, food consumption, hematological profile, and histology^12–17^. Thus, gallic acid holds considerable promise as a candidate anti-cancer agent with low side effects. The need for novel treatments persists, due to established chemotherapy and radiotherapy frequently causing severe side effects, such as cardiotoxicity and blood disorders, which can lead to treatment failure^18–20^.

One of the most important therapeutic strategies for cancer treatments is activating apoptotic pathways^21^. We and other groups have demonstrated that in human cancer cell lines, gallic acid can trigger robust apoptosis, characterized by hallmarks of apoptosis, such as cytochrome *c* release from mitochondria to cytosol, caspase-3 activation, nuclear condensation, cell shrinkage, and plasma membrane blebbing^11,22^. Recently, we further demonstrated that gallic acid actually triggers an iron-dependent cell death process, with apoptotic, ferroptotic, and necroptotic features^22^. For example, administration of the iron chelator deferoxamine (DFO) can efficiently block initiation of gallic acid-induced cell death, suggesting its link to ferroptotic cell death pathway^22^. In addition, extensive plasma membrane swelling and rupture were observed shortly after caspase-3 activation, suggesting the involvement of necroptosis in this cell death process^22^.

To harness the discovery of the anti-cancer property of gallic acid for developing a new therapeutic intervention, it is essential to understand how gallic acid mediates cell demise. Elucidating a functional mechanism requires our understanding of global gene expression of the corresponding cellular process. In this pursuit, we conducted a time-course study to evaluate the global gene expression profile at the early, middle, and late stages of gallic acid-induced cancer cell death, using high-throughput transcriptome-wide analysis of differential gene expression with RNA-sequencing technology. As gallic acid triggers cell death with apoptotic, ferroptotic, and necroptotic features, its mechanism is likely to be complex. Here, we found striking changes in gene transcription across all three stages, including the early enrichment of ferroptosis-related pathways, followed by enrichment of apoptotic and lysosomal pathways, and later the enrichment of necroptosis-related pathways. Our study provides important insights into the activation of multiple cell death process by gallic acid in cancer cells.

## Results and discussion

### Validation of HeLa cells as a suitable in vitro study model for gallic acid-induced cell death

**Figure 1:**
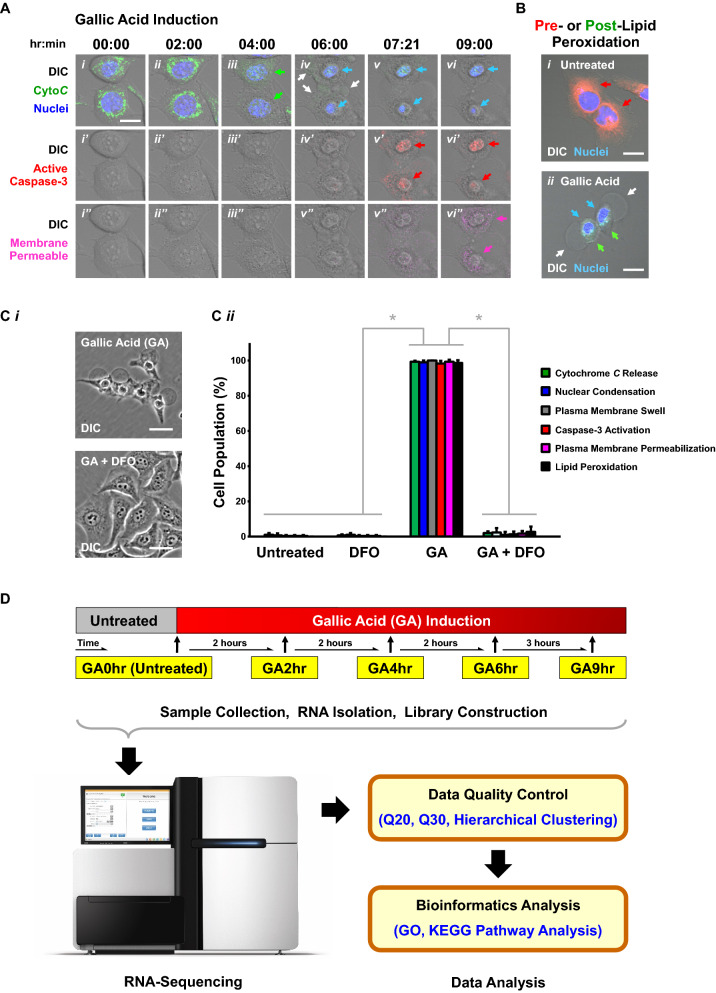
Overview of experimental design. (**A**) Model of gallic acid-induced cell death in human cervical cancer HeLa cells. Time-lapse live-cell confocal microscopy of the same group of cytochrome *c*-GFP expressing HeLa cells after treatment with 50 µg/mL of gallic acid. (**i**–**vi**) Top row, merged images of differential interference contrast (DIC) microscopy, cytochrome *c*-GFP, and nucleus; (**i′**–**vi′**) Middle row, DIC and caspase red substrate; (**i′′**–**vi′′**) Bottom row, DIC and plasma membrane-permeable dye. Arrows: blue, nuclear condensation; green, cytochrome *c* release from mitochondria to cytosol; pink, plasma membrane permeabilization; red, caspase-3 activation; white, plasma membrane swelling. Scale bar: 10 µm. (**B**) Apoptotic, ferroptotic, and necroptotic features displayed in gallic acid-induced HeLa cells. Confocal images of (**i**) Untreated HeLa cells, and (**ii**) HeLa cells treated with gallic acid (50 µg/mL) for 24 h. The HeLa cells were stained with Hoechst 33342 for nuclear morphology, and Image-iT lipid peroxidation sensor to detect pre-lipid peroxidation (red) and post-lipid peroxidation (green) of the cells. DIC microscopy was performed to observe cell morphology. Arrows: blue, nuclear condensation; green, post-lipid peroxidation; red, pre-lipid peroxidation; white, plasma membrane swelling. Scale bar: 5 µm. (**C**) Suppression of gallic acid-induced cell death by the iron chelator deferoxamine. (**i**) Phase contrast images of HeLa cells treated with gallic acid (GA, 50 µg/mL) alone or with co-treatment with inhibitor deferoxamine (DFO, 200 µM) for 24 h. Scale bar: 30 µm. (**ii**) Quantification of the cell death events, including cytochrome *c* release, nuclear condensation, plasma membrane swell, caspase-3 activation, plasma membrane permeabilization, and lipid peroxidation, in HeLa cells at 24th hour after treatment of 50 µg/mL gallic acid alone (GA) or co-treatment of gallic acid with inhibitor 200 µM DFO (GA + DFO). Untreated cells (Untreated) or cells treated with DFO alone (DFO) serve as controls. Mean ± s.d.; n = 3. The data was evaluated using one-way analysis of variance (ANOVA) followed by Tukey's test for post-hoc analysis. *p < 0.01. (**D**) Schematic for experimental design and procedures. HeLa cells were treated with 50 µg/mL gallic acid, and where then collected at 2 h (GA2hr), 4 h (GA4hr), 6 h (GA6hr), and 9 h (GA9hr) after incubation with gallic acid. At each time point, three samples were collected. The cells without gallic acid treatment served as the control (GA0hr). The samples were then subjected to RNA isolation, library construction, RNA-sequencing, and bioinformatics analysis, to determine the gene expression profile of the cells at different stages of the gallic acid-induced cell death process.

We began this study by validating our in vitro model and condition of cell death induction (Fig. 1). Studies demonstrated gallic acid as a potent cell death inducer to various human cancer cell lines^11^. In our present study, we selected human cervical cancer HeLa cells as our model, because this cell line is one of the most commonly used in vitro model for gallic acid research^11^, and is widely used in biomedical research particularly in cancer biology^23,24^.

For cell death induction, we used the physiological relevant concentration of 50 µg/mL of gallic acid because of 3 major reasons. (1) In vivo studies showed that that administration of this dosage of gallic acid is well-tolerated without adverse side effect, and it displays anti-cancer effects in rodent models^12–17^. (2) This dosage is commonly used in in vitro studies for characterizing the anti-cancer property of gallic acid^11,25,26^. (3) This dosage triggers cell death with apoptotic, ferroptotic, and necroptotic features in our current model of gallic acid-induced HeLa cells (Fig. 1), as well as other human cancer cell lines as we recently reported^22^.

We have first verified our model by time-lapse live-cell imaging (Fig. 1A, Video S1). To begin, the HeLa cells stably expressing cytochrome *c*-GFP were stained with Hoechst 33342 to visualize the nucleus. These cells were then incubated in cell culture medium with NucView 530 Caspase-3 substrate to detect activation of caspase-3 (labeled by red fluorescence), and with IncuCyte Cytotox red dye to detect plasma membrane rupture (labeled by deep red “pink” fluorescence in cells with plasma membrane-permeabilization).

Before application of gallic acid to the cell culture medium, the HeLa cells had a round nucleus, and were flat, spreading their cytoplasm on the substrate, (Fig. 1Ai). The cytochrome *c*-GFP was located in the mitochondria, displaying a network structure in these untreated cells (Fig. 1Ai). At the 2nd hour of gallic acid induction, there was no hallmark of cell death in the cells observed, so that this time point was considered at the early stage of the cell death process (Fig. 1Aii). At the later time points of the gallic acid induction, as expected^11,22^, the HeLa cells displayed the hallmarks of apoptosis, evidenced by: (1) the release of cytochrome *c*-GFP from the mitochondria to cytosol at 4th hour after the gallic acid induction (Fig. 1Aiii, *green arrows*), (2) nuclear condensation starting at the 6th hour, (Fig. 1Aiv–vi, *blue arrows*), and (3) caspase-3 activation starting at 7.3th hour (Fig. 1Av′–vi′, *red arrows*). These observations confirm that gallic acid triggers the apoptotic cell death pathways in HeLa cells.

Interestingly, our recent study also suggested the induction of additional cell death pathways by gallic acid^22^, as shown by our time-lapse live-cell imaging here. During apoptosis, dying cells should display morphological changes of cell shrinkage^27,28^. However, gallic acid-induced dying cells displayed plasma membrane swelling started at the 6th hour after gallic acid induction (Fig. 1Aiv, *white arrows*), and plasma membrane rupture at the 9th hour after gallic acid induction (Fig. 1Avi′′, *pink arrows*). These are the hallmarks of necrosis or necroptosis^29,30^, contrasting with cell shrinkage characteristic of apoptosis^28^. Furthermore, the plasma membrane rupture (Fig. 1Avi′′, *pink arrows*, at 9th hour) was an acute process that occurred shortly after the caspase-3 activation (Figs. 1Av′ and S1ii′–iii′, ii′′–iii′′, *red arrows*, at 7.3th hour). This observation suggests that loss of plasma membrane integrity was mediated by an active process, such as necroptosis^22^. This is a further contrast with apoptotic cells which are characterized by maintenance of plasma membrane integrity^28,31–33^. Even if loss of plasma membrane integrity could occur due to a gradual and passive process namely “secondary necrosis” in apoptotic cells, this can take hours, days or even longer^34,35^; thus our observation of plasma membrane rupture occurring shortly after caspase-3 activation in the gallic acid induction contrasts with apoptosis and secondary necrosis.

In addition, our previous study revealed that the gallic acid-induced cell death process involves activation of ferroptosis in HeLa cells^22^, as indicated by lipid peroxidation (Fig. 1B*, green arrows*), which is the hallmark of ferroptosis^36–38^. Importantly, these gallic acid-induced cells also displayed nuclear condensation (Fig. 1B, *blue arrows*) as the hallmark of apoptosis^28,33^, and plasma membrane swelling (Fig. 1B, *white arrows*) as the hallmark of necroptosis^29,30^. These indicate that HeLa cells underwent apoptosis, ferroptosis, and necroptosis after induction of gallic acid (Fig. 1B)^22^.

Ferroptosis is an iron-dependent cell death process^36–38^, which can be suppressed by iron chelators, such as deferoxamine (DFO)^36–38^. Interestingly, we found that DFO efficiently suppressed gallic acid-induced cell death, when DFO and gallic acid were applied together to treat the cells (Figs. 1Ci–ii and S2)^22^. This indicates the involvement of ferroptosis in the initiation of the gallic acid-induced cell death process.

Taken together, our experiments validated that gallic acid-induced HeLa cells display apoptotic, ferroptotic, and necroptotic features (Fig. 1A–C). Therefore, we will use this in vitro model to reveal the molecular signature of gallic acid-induced cell death process in this study.

### Experimental design and workflow of RNA-sequencing

To develop a new anti-cancer therapy with the use of gallic acid, the first critical step is to understand the molecular mechanism governing this cell death process. RNA-sequencing is an important technology to understand the complexity of phenotypes and biological pathways, and to investigate regulation of both normal functions and diseases^39–41^. Therefore, we applied RNA-sequencing to determine the gene expression profile at four time points after gallic acid induction, 50 µg/mL, to HeLa cells: 2nd hour (early stage), 4th hour (first middle stage), 6th hour (second middle stage), and 9th hour (late stage). The untreated cells (0 h) served as the control (Fig. 1D).

We selected the 2nd hour to represent the “early stage” of gallic acid-induced cell death process, as we did not detect any hallmark of cell death at this time point (Fig. 1Aii). The 4th hour was selected as the “first middle stage”, because the gallic acid-induced cells only displayed cytochrome *c* release, the first hallmark of cell death that was detected (Fig. 1Aiii). The 6th hour was the “second middle stage”, as the induced cells displayed not only cytochrome *c* release, but also nuclear condensation and cytoplasmic swelling (Fig. 1Av). The 9th hour was the “late stage” of the cell death process, as the induced cells further displayed caspase-3 activation (Fig. 1Av′) and plasma membrane permeabilization (Fig. 1Avi′′).

We collected RNA, with three replicates at each of the five time points, giving a total of 15 samples, and performed RNA-sequencing (Table S1). We adopted the Illumina HiSeq 4000 platform to perform our RNA-sequencing, as this is one of the most reliable and commonly used next generation sequencing platforms commercially available for biological research^39–41^. We conducted the sequencing with the paired-end 150 base pairs as the sequencing approach. We performed quality control of the raw RNA-sequencing data, before subjecting it to bioinformatics data analysis (Fig. 1D).

### Quality control of raw RNA-sequencing data

Table S1 summarizes the statistics of our data obtained from the RNA-sequencing, and shows that we collected high quality data.

We first looked at the number of read pairs, which indicated the read depth of our sequencing data (Table S1). For each of our 15 samples, we obtained 12 gigabyte (G) of raw data per sample. The corresponding numbers of read pairs ranged from 57.7 to 74.7 million reads. After data filtering and mapping to a reference genome, we have obtained “clean” data, with number of bases ranging from 8.72 to 11.29G per sample. This indicated that the total numbers of sequencing reads obtained from each of our sample had high read depth (Table S1), which exceeded the general standard that requires 5G of data for data analysis of RNA-sequencing^39,40,42^.

We then examined the quality of the data that we obtained, by using the Phred quality score (Q score), which is the most common protocol used to indicate the accuracy of reads, and determine the quality of data obtained by a sequencer^40,43,44^. For example, the Phred Quality Score of 20 (Q20) means the possibility of an incorrect base call by the sequencer is 1 in 100 times, which means the probability of a correct base call by the sequencer is 99%. For the Phred Quality Score of 30 (Q30), it means the possibility of an incorrect base call by the sequencer is 1 in 1000 times, indicating that the probability of a correct base call by the sequencer is 99.9%. In our present study, after mapping to a reference genome, all of our samples had Q20 scores > 96.38% (ranging from 96.38 to 97.21%). Importantly, all the Q30 scores were > 92.28% (ranging from 92.28 to 93.56%). This Q30 score is significantly higher than the current official standard from Illumina (Q30 score ≥ 80%). Furthermore, the overall alignment rate for all samples was ≥ 71.71% (ranging from 71.71 to 85.29%). In summary, our RNA-sequencing yielded high-quality data for analysis (Table S1).

### Technical validation of RNA-sequencing data set via a heat map

A common practice for gaining an overview of samples is visualizing the gene expression profile of the data set via a heat map^45,46^. By combining the heatmap and the clustering methods, the samples within the data set can be compared, based on their similarity and difference in their corresponding gene expression profiles^45,46^.

**Figure 2:**
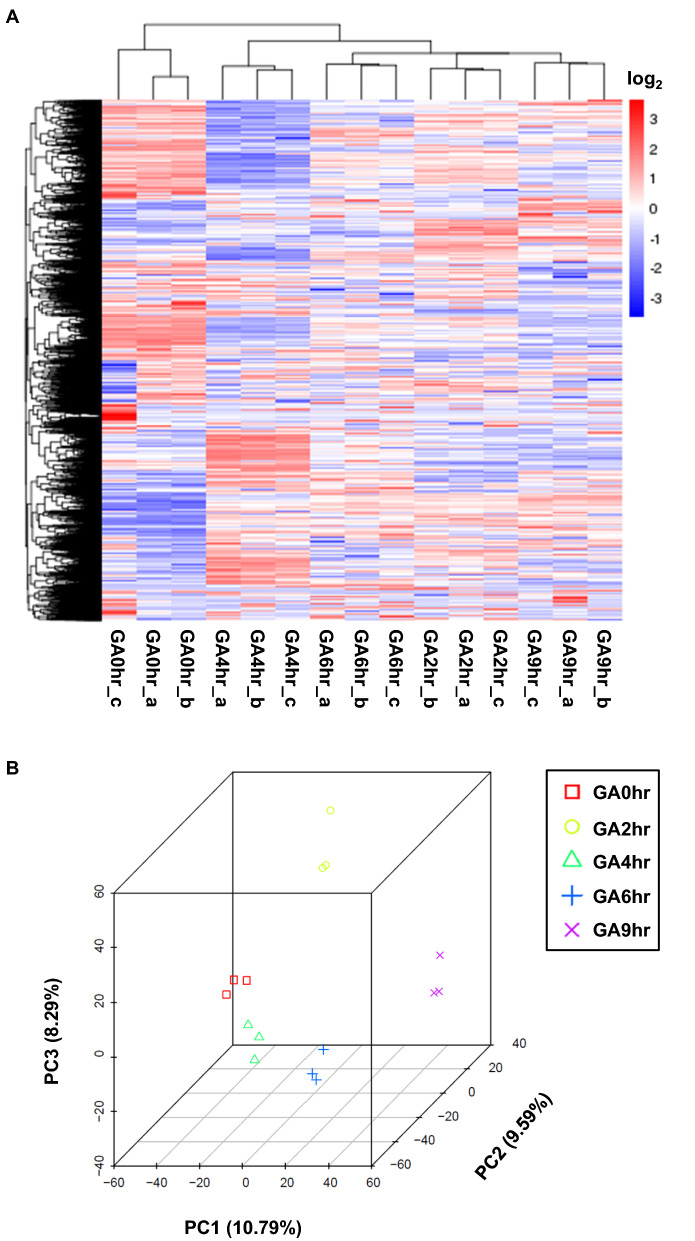
Technical validation of RNA-sequencing data set. The three biological replicate samples (a, b, c) of RNA-sequencing data are shown to cluster together by using (**A**) unsupervised hierarchical clustering, and (**B**) principal component analysis (PCA) of the 15 samples, three each from the 0th hour (GA0hr), 2nd hour (GA2hr), 4th hour (GA4hr), 6th hour (GA6hr) and 9th hour (GA9hr) of the gallic acid-induced cell death process. The corresponding summary of the data set is listed in Table S1.

Therefore, we applied unsupervised hierarchical clustering to generate a heat map to examine our data set (Fig. 2A). This approach adopts unsupervised pattern recognition, applying unsupervised machine-learning techniques to identify the patterns inherent in a data set^47^. This unsupervised algorithm aims to compare the gene expression profiles between samples, and to group them based on the similarity of the “patterns” shown, according to the level of the expressions of the genes in each sample.

In the heat map generated by the unsupervised hierarchical clustering with our data set (Fig. 2A), the columns (X-axis) list all of the 15 samples in the data set. The rows (Y-axis) list the genes that were identified by the RNA-sequencer. The level of the expression of each gene is “color-coded”, with red representing up-regulation of gene expression, and blue representing down-regulation of gene expression. The level of the gene expression, as represented by this color code, is shown as the log_2_ scale indicated in the scale bar in the Fig. 2A.

In the heat map, the hierarchical clustering arranges: (1) the orders, and ranks of the samples in the columns, according to the similarity in expression of the same genes, and (2) the orders of the genes in the rows, according to their similarity in their corresponding expression. The hierarchical dendrogram in the heat map is an important tool to illuminate the structure between, and within, clusters of the samples^42,45–47^. The dendrogram in the columns indicates the similarity between each sample based on their gene expression profile of the same genes, while the dendrogram in the rows indicate the genes with the similar expression patterns. The shorter the “branch” between any two points, the higher is their corresponding correlation (similarity), and vice versa.

Therefore, our heat map of unsupervised hierarchical clustering reveals the similarity between all the replicates at each time point (Fig. 2A). First of all, all corresponding replicates in each of the time points are grouped together by the unsupervised algorithm. The dendrogram further confirms this observation by connecting the replicates in the same time point with the shortest branches. This result indicates that the replicates belonging to the same time point had the highest correlation, according to their highest similarity with the replications, and also the difference the samples from different time points.

Importantly, the dendrogram at the column also indicated that the samples from the gallic acid treatment for 2 h, 4 h, 6 h, and 9 h (GA2hr, GA4hr, GA6hr, GA9hr) share certain different correlation in the gene expression profile, therefore indicating that different genes (cellular mechanisms) were activated at the different stages of the gallic acid-induced cell death (Fig. 2A). This has been verified at the later part of this study. Interestingly, the dendrogram further shows that all of these gallic acid-induced cells display expression that is distinct from the control cells that did not receive gallic acid treatment (GA0hr). This finding indicates that gallic acid-induced cell death is regulated by an active and unique mechanism that is quite different from the normal biological process (Fig. 2A).

### Technical validation of RNA-sequencing data set by principal component analysis

We further validated the replicates of our RNA-sequencing data set by using principal component analysis (PCA) (Fig. 2B). PCA is a mathematical algorithm that can reveal key features and relationships of dimensionality of the data set^48,49^. Application of PCA includes determination of clustering of gene expression between samples in a data set, thereby allowing comparison of the relationship among samples for their similarity and difference. Samples are calculated and scored according to features of the expression profiles of their individual genes, and are presented according to their dimensionality, which can be allocated as vectors in the X-, Y-, and Z-axes. Thus, we can visualize the similarities and differences between replicates in each time points during the induction of gallic acid, and determine their relationships according to distance between the corresponding samples in the three-dimensional plot.

Here, the three-dimensional plot of the principal component analysis (3DPCA) demonstrated agreement among the biological replicates (Fig. 2B), in agreement with the unsupervised hierarchical clustering (Fig. 2A). In the 3DPCA plot, the individual samples that belong to the same time point are labeled with the same color, while the individual samples belonging to different time points are identified with different colors. The 3DPCA plot shows that all the biological replicates that belong to the same time point form close clusters (Fig. 2B). This finding reveals the high similarly of the gene expression profile between the biological replicates, indicating that our data is highly repeatable between the replicates. Furthermore, there are the distinct loci for each cluster from different time points, which indicate the differences in the gene expression profiles between different time points of the samples (Fig. 2B).

### Validation of RNA-sequencing data by reverse transcription polymerase chain reaction

We validated our RNA-sequencing data by using reverse transcription polymerase chain reaction (RT-PCR) in human cervical cancer HeLa cells. We randomly selected four genes that displayed differential expression in response to gallic acid induction, and designed specific primers to detect their expression level (Table S2). The two housekeeping genes, RPL13AP3 and UBC, which did not display change in their expression level in our RNA-sequencing data, served as controls.

Our RT-PCR confirmed that the four genes displayed the expected changes in expression in the gallic acid-induced HeLa cells, comparing with the untreated HeLa cells (Fig. S3). At the same time, the expression of the housekeeping genes remained at the same level in the gallic acid-induced and the untreated cells. Importantly, we also confirmed these results in 3 different human cancer cell lines, including melanoma A-375 cells, osteosarcoma MG-63 cells, and bladder carcinoma 5637 cells (Fig. S3). In summary, our RT-PCR results validated our RNA-sequencing data in the same and different human cancer cell lines.

### Gene expression profile of gallic acid-induced cell death

**Figure 3:**
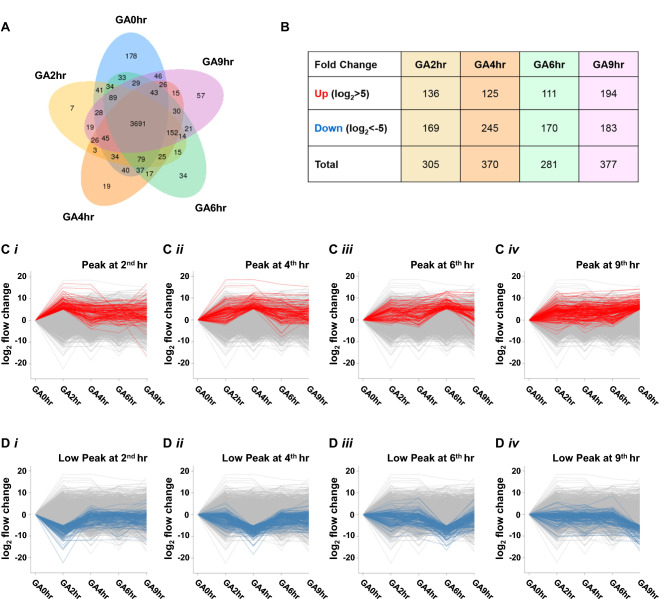
Change of gene expression profiles during gallic acid-induced cell death in HeLa cells. (**A**) Venn diagram reveals the number of common or unique genes that were differentially expressed (FPKM > 1) at five time points of the gallic acid-induced cell death process. (**B**) Number of up-regulated or down-regulated genes at the 2nd hour (GA2hr), 4th hour (GA4hr), 6th hour (GA6hr) and 9th hour (GA9hr) of the gallic acid-induced cell death process. (**C**) Up-regulation of gene expression during the gallic acid-induced cell death process. Log_2_ fold change of gene expression at the (**i**) 2nd hour (GA2hr), (**ii**) 4th hour (GA4hr), (**iii**) 6th hour (GA6hr) and (**iv**) 9th hour (GA9hr) of the gallic acid-induced cell death process. The genes that display the highest expression (peak), together with log_2_ fold change > 5, at the corresponding time point are highlighted in red. The log_2_ signal values from three biological replicates were averaged for each time point, and normalized with the untreated cells (GA0hr). (**D**) Down-regulation of gene expression during the gallic acid-induced cell death process. Log_2_-fold change of gene expression at the (**i**) 2nd hour (GA2hr), (**ii**) 4th hour (GA4hr), (**iii**) 6th hour (GA6hr) and (**iv**) 9th hour (GA9hr) of the gallic acid-induced cell death process. The genes that display the lowest expression (low peak), together with log_2_ fold change > 5 at the corresponding time point are highlighted in blue. The log_2_ signal values from three biological replicates were averaged for each time point, and normalized with the untreated cells (GA0hr).

To reveal the molecular signature of gallic acid-induced cell death, we first identified the genes differentially expressed in the four time point treated samples (GA2hr, GA4hr, GA6hr, GA9hr) relative to the untreated sample (GA0hr). By generating a Venn diagram, we further compared all the expressed genes with FPKM (Fragments Per Kilobase Million) in all the samples, in order to identify their unique and common differential expression transcripts among each compared time points during the gallic acid-induced cell death process. The Venn diagram reveals differentially expressed genes in all samples (Fig. 3A). At each of the four time points after gallic acid induction, an increasing number of genes displayed unique expression: 7 genes at GA2hr, 19 genes at GA4hr, 34 genes at GA6hr, and 57 genes at GA9hr. These genes were not expressed in the control (untreated, GA0hr) cells (Fig. 3A).

We then determined which genes displayed the highest expression (up peak) and lowest expression (low peak) at each of the time points (stages) of the gallic acid-induced cell death process (Fig. 3B). We found for each of the time points, that the number of genes that were up-regulated (fold change log_2_ > 5) and down-regulated (fold change log_2_ < − 5) respectively, compared with the untreated sample (GA0hr) (Fig. 3B) were: GA2hr 136 and 169 (Tables S3 and S4), GA4hr 125 and 245 (Tables S5 and S6), GA6hr 111 and 170 (Tables S7 and S8), and GA9hr 194 and 183 (Tables S9 and S10).

We also visualized the change of gene expression across different time points of the gallic acid-induced cell death process (Fig. 3C, D). The X-axis of each plot in the corresponding figures lists the samples. The Y-axis of the plots indicates the log_2_ value of the fold change of the genes, with a positive value indicating up-regulation (Fig. 3C, red), and a negative value indicating down-regulation (Fig. 3D, blue). Each line in the plot represents the expression of a gene. For each time point, the log_2_ signal values of each gene from three biological replicates were averaged. To determine the change of the gene expression profile with time during gallic acid induction, all values of the expression level were compared with the level of the untreated cells (GA0hr).

Specifically, Fig. 3C presents plots that highlight genes displaying the highest expression at GA2hr (Fig. 3Ci, Table S3), GA4hr (Fig. 3Cii, Table S5), GA6hr (Fig. 3Ciii, Table S7), and GA9hr (Fig. 3Civ, Table S9), while Fig. 3D presents corresponding data for genes displaying the lowest expression at GA2hr (Fig. 3Di, Table S4), GA4hr (Fig. 3Dii, Table S6), GA6hr (Fig. 3Diii, Table S8), and GA9hr (Fig. 3Div, Table S10). These plots reveal different gene expression pattern at the four different time points of the cell death process, suggesting the activation of different biological processes. Taken together, our data indicates that gallic acid triggers a transcriptionally active cell death process.

### Pathways analysis of gallic acid-induced cell death

To understand the molecular mechanism of gallic acid-induced cell death, we performed bioinformatics analysis to study which biological pathways and functions could be mediated by the differentially expressed genes identified at the four time points in the cell death process.

**Figure 4:**
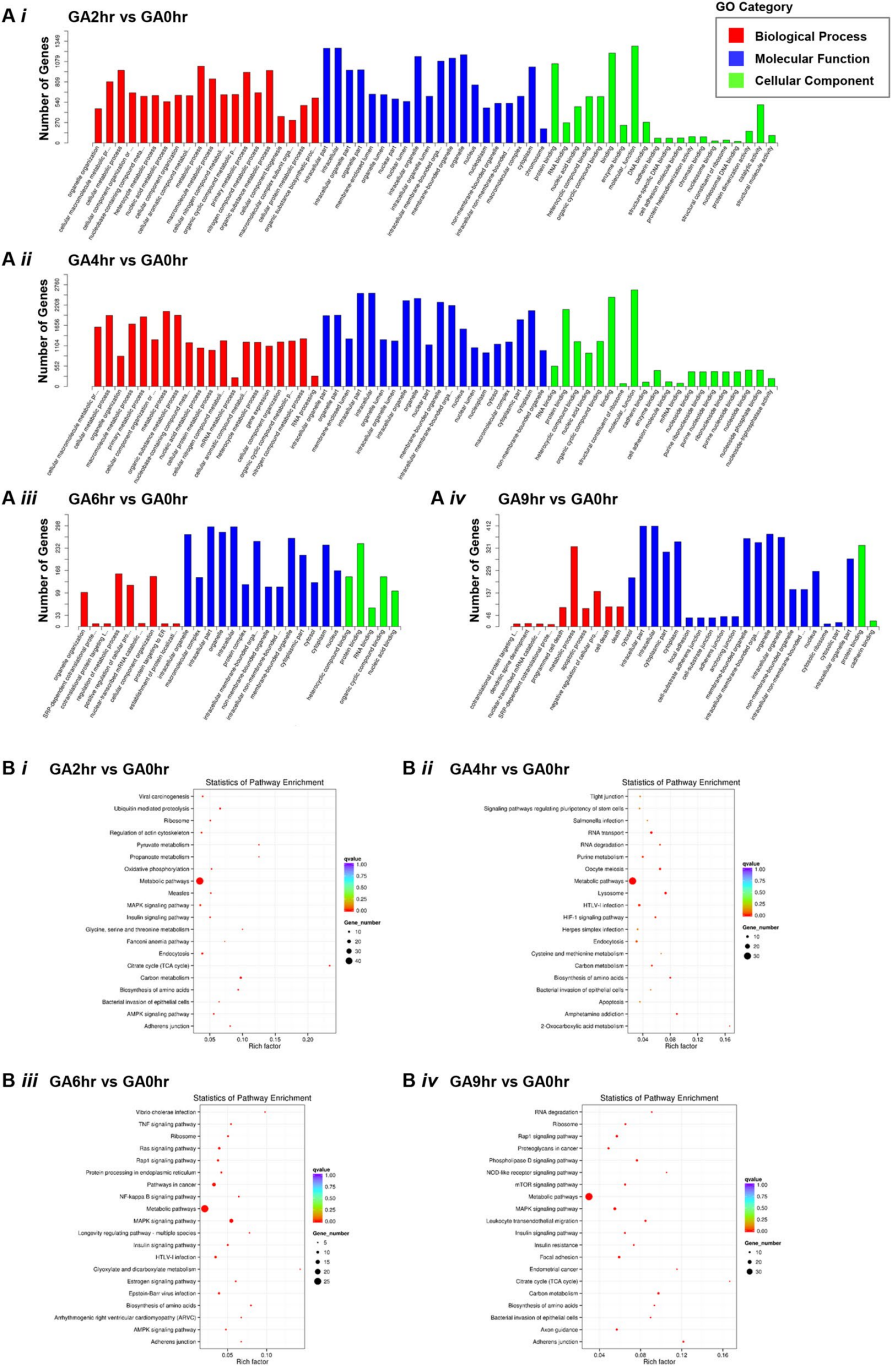
Time-course GO and KEGG analysis of the gallic acid-induced cell death process. (**A**) Gene Ontology (GO) classification, and (**B**) Kyoto Encyclopedia of Genes and Genomes (KEGG) pathway enrichment analyses of the differentially expressed genes at the (**i**) 2nd hour (GA2hr), (**ii**) 4th hour (GA4hr), (**iii**) 6th hour (GA6hr) and (**iv**) 9th hour (GA9hr) of the gallic acid-induced cell death process.

We first performed the Gene Ontology (GO) enrichment classification analysis (Fig. 4A). The GO is a bioinformatics approach for determining which biological processes, molecular functions and cellular components correlated with a list of genes identified such as by genomic sequencing^50^. We found that there were 20 enrichments in GA2hr and GA4hr in each of the three categories of biological processes, molecular functions and cellular components (Fig. 4Ai, ii). At GA6hr, there were only 9 enrichments of biological process, 14 enrichments of molecular function, and 5 enrichments of cellular components (Fig. 4Aiii). At GA9hr, there were only 10 enrichments of biological process, 20 enrichments of molecular function, and 2 enrichments of cellular component identified in GA9hr (Fig. 4Aiv).

We further performed Kyoto Encyclopedia of Genes and Genomes (KEGG) enrichment analysis on our data set (Fig. 4B). The KEGG is an online database resource for a knowledge-based systematic analysis of gene functions, which aims to link genomic information with higher order functional information^51,52^. The results of the KEGG analysis are shown as a graphical representation of the scatter plots (Fig. 4B). Each figure represents KEGG enrichment of the top 20 identified pathways for each of the time points, GA2hr (Fig. 4Bi), GA4hr (Fig. 4Bii), GA6hr (Fig. 4Biii), GA9hr (Fig. 4Biv), with corresponding rich factor, q-value, and the number of the enriched genes in the corresponding pathways. The rich factor in the scatter plots is defined as the number of the enriched candidate genes versus the total number of the annotated genes (number of enriched genes/ numbers of total annotated genes) that are considered by the KEGG analysis in the corresponding pathway. Therefore, a higher rich factor suggests the more significant enrichment of the candidate genes in the corresponding pathway. The q-value shown in the KEGG graph is the adjusted *p*-value, which indicates the false discovery rate for measuring variables of a large data set, such as for the level of gene expression of RNA-sequencing data.

According to the GO and KEGG analysis, we have identified multiple pathways that could directly or indirectly contribute in the early (GA2hr), middle (GA4hr, GA6hr), and late (GA9hr) stages of the gallic acid-induced cell death process.

### Second hour of gallic acid induction: enriched pathways

At the 2nd hour (GA2hr) of the gallic acid induction (Fig. 4Ai, Bi), we identified enrichment of the “metabolic pathways”, “citrate cycle (TCA cycle)”, and “biosynthesis of amino acids”. This finding suggests that gallic acid-induced cell death is a metabolically active cell death process. We identified the “AMPK signaling pathway” (Fig. 4Bi). This is in agreement with previous studies that demonstrated the activation of AMPK pathway by gallic acid^53,54^. It is also known that AMPK pathway plays an important role in coordinating the signals that mediate metabolisms^55^. Therefore, our findings on the enrichment of the AMPK pathway supports our observation on the activation of the multiple metabolic pathways by gallic acid in the present study.

Interestingly, we also identified enrichment of “oxidative phosphorylation” (Fig. 4Bi), which can contribute to the generation of reactive oxygen species (ROS), creating the oxidative cellular environment for the execution of ferroptosis^56^. This is in agreement with our RNA-sequencing data, which showed the homeostatic iron regulator (HFE) to display over a 3.8 log_2_ fold change at the second hour of the gallic acid induction (Fig. 3Ci), thereby revealing its link to the activation of the iron-dependent ferroptotic cell death. This observation also agrees with our observation that gallic acid is an iron-dependent cell death process, which can be inhibited by the iron chelator deferoxamine (DFO) (Figs. 1C and S2)^22^.

Besides, we have identified the enrichment of pathways for initiating cell death. These include “AMPK signaling pathway” as mentioned above, and also “MAPK signaling pathway” at the 2nd hour (GA2hr) of the gallic acid induction (Fig. 4Ai, Bi). Previous studies have demonstrated that “AMPK signaling pathway” can contribute to cell death such as apoptosis due to its direct or indirect interactions with AKT^55^. The “MAPK signaling pathway” plays critical role in mediating apoptotic^57^ and ferroptotic^58^ cell death. Therefore, the AMPK and MAPK signaling pathways could mediate the early stage of gallic acid-induced cell death process.

### Fourth hour of gallic acid induction: enriched pathways

At the 4th hour (GA4hr) of the gallic acid induction (Fig. 4Aii, Bii), multiple metabolism-related pathways were enriched: “metabolic pathways”, “purine metabolism”, “carbon metabolism”, “biosynthesis of amino acids”, and “2-oxocarboxylic acid metabolism”. These are in agreement with the active metabolism identified at the 2nd hour of the gallic acid induction, indicating that gallic acid-induced cell death is an active cell death process. The “RNA transport” pathway was also identified, suggesting the involvement of active translation and transcription^59,60^.

Notably, at the fourth hour, we identified enrichment of “apoptosis” and the “lysosome” pathways (Fig. 4Bii), which can mediate the activation of apoptosis by triggering caspase activation^61^, the hallmark of apoptosis^62^. This observation is supported by our raw RNA-sequencing data, which showed that the mRNAs that encode caspases displayed over 5 log_2_ fold change (Fig. 3Cii). This result is also supported by our time-lapse live-cell imaging which showed that caspase-3 activation to occur during the gallic acid induction (Fig. 1Av′–vi′)^22^. Importantly, the activation of the “lysosome” pathways can trigger ferroptosis^63^ and necroptosis^64^. Taken together, these findings reveal the link of gallic acid-induced cell death with the activation of ferroptosis as described above, and also necroptosis at the sixth hour time point as described below.

### Sixth hour of gallic acid induction: enriched pathways

At the 6th hour (GA6hr) of gallic acid induction (Fig. 4Aiii, Biii), the “metabolic pathways” and “biosynthesis of amino acid” pathways were enriched, as were the cases at second and forth hours. Multiple cell signaling pathways were also enriched, including “Ras signaling pathway”, “MAPK signaling pathway”, “estrogen signaling pathway”, and the “AMPK signaling pathway”. These signaling pathways are closely related to the regulation of cell death and survival, and are the pathways for therapeutic targeting of cancer cells^65–68^. These findings explain the identification of the “pathways in cancer” (Fig. 4Biii).

Importantly, at the sixth hour time point, we also identified the “TNF signaling pathway” (Fig. 4Biii), which suggests activation of the necroptotic cell death pathway by gallic acid. Supporting this contention, our RNA-sequencing analysis revealed up-regulation of genes that contribute to necroptosis, such as the mRNAs that encode tumor necrosis factor (log_2_ > 2.9) and caspase-8 (log_2_ > 3.5) (Fig. 3Ciii). This finding agrees with our observation that gallic acid-induced cancer cells display the hallmarks of necroptosis such as plasma membrane swelling and permeabilization (Fig. 1Aiv, vi′′) during the gallic acid-induced cell death process^22^.

### Ninth hour of gallic acid induction: enriched pathways

At the 9th hour (GA9hr) of the gallic acid induction (Fig. 4Aiv, Biv), we identified the enrichment of “apoptotic process”, “cell death”, “death” “programmed cell death”, and “RNA degradation”, indicating destruction of the cells. This finding is in agreement with our observation, by time-lapse live-cell microscopy, that this is the terminal stage of the cell death process, as evidenced by nuclear condensation, caspase-3 activation, and plasma membrane permeabilization (Fig. 1Avi–vi′′)^22^.

We have identified the enrichment of “metabolic pathways”, “biosynthesis of amino acid”, “carbon metabolism”, “citrate cycle”, “MAPK signaling pathway”, in this (Fig. 4Biv) and also the earlier time points as described above. At the 9th hour, we further identified ‘mTOR signaling pathway” (Fig. 4Biv), which can contribute to apoptosis^69,70^. These observations suggest that gallic acid triggered a metabolically active cell death process, even at the late stage.

### Importance of amino acid and iron metabolism in executing gallic acid-induced cell death

**Figure 5:**
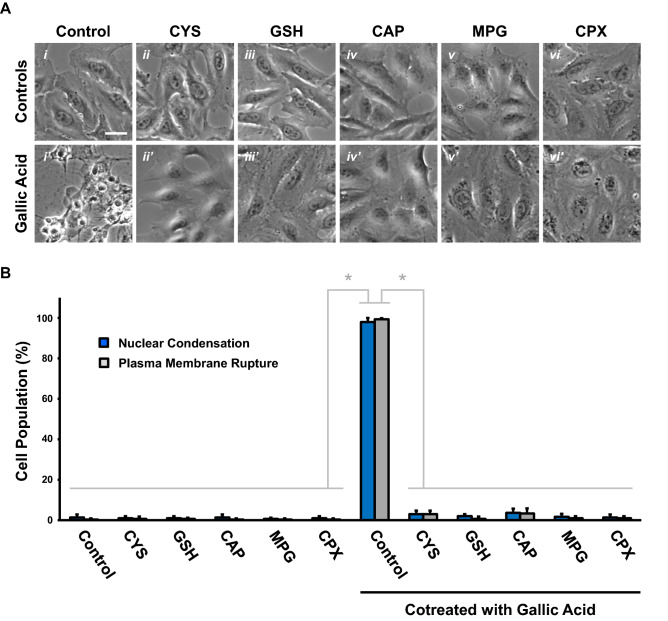
Inhibitors of gallic acid-induced cell death. (**A**) Phase contrast images of HeLa cells preincubated with (**i**) medium alone (Control), (**ii**) cysteine (CYS, 5 mM), (**iii**) glutathione (GSH, 10 mM), (**iv**) captopril (CAP, 10 mM), (**v**) N-(2-mercaptopropionyl)-glycine (MPG, 3 mM), or (**vi**) ciclopirox (CPX, 5 µM), for 1h and then with (lower panels, (**i**′–**vi**′)) or without (upper panels, (**i**–**vi**)) co-treatment of gallic acid (50 µg/mL) for 9 h. Scale bar: 30 µm. (**B**) Quantification of the cell death events, including nuclear condensation (blue) and plasma membrane rupture (grey), in HeLa cells after gallic acid induction (50 µg/mL) with or without co-treatment with compounds with the conditions listed at the panel A for 9 h. Mean ± s.d.; n = 3. The data was evaluated using one-way analysis of variance (ANOVA) followed by Tukey's test for post-hoc analysis. *p < 0.01.

Our RNA-sequencing data revealed the regulator candidates for the initiation and execution of gallic acid-induced cell death. By using the small molecules to target these regulators and their pathways (Fig. 5), we identified the key regulators for controlling this cell death process.

Our bioinformatics analysis indicated the enrichment of “biosynthesis of amino acid” at the 2nd, 4th, 6th, and 9th hour of gallic acid induction (Fig. 4). As we found gallic acid is an iron-dependent cell death process with ferroptotic features^22^, and the initiation and execution of ferroptosis are tightly linked with amino acid metabolism^36,37,71,72^, we first tested their association with gallic acid-induced cell death process.

Lipid peroxidation is the hallmark of ferroptosis^36,37^. To maintain normal cellular redox homeostasis, cystine is reduced to cysteine (CYS) for the use of synthesizing glutathione (GSH), which serves as a cofactor for the selenoenzyme glutathione peroxidase 4 (GPX4) for protecting cells by eliminating lipid peroxides^71–73^.

We were curious if the initiation of gallic acid-induced cell death was mediated by the CYS and GSH biosynthesis and GPX4 pathways. If true, gallic acid-induced cell death should be suppressed by the supplement of CYS and GSH. To test this hypothesis, we therefore preincubated HeLa cells with CYS (5 mM) or GSH (10 mM) for an hour, and then co-treated the cells with gallic acid (50 μg/mL). The cells treated with CYS or GSH alone served as controls.

We found that HeLa cells treated with CYS or GSH alone did not show any signs of cell death and were comparable to the untreated cells (Fig. 5Ai–iii, B). Interestingly, co-treatment of CYS or GSH with gallic acid did suppress gallic acid-induced cell death (Fig. 5Ai′–iii′, B). This indicates CYS and GSH biosynthesis and GPX4 pathways are important for the initiation of gallic acid-induced cell death process.

Depletion of amino acid biosynthesis and GPX4 pathways can lead to oxidation stress to the cells^36,37,71,72^. Our bioinformatics analysis also indicated the enrichment of “oxidative phosphorylation” at the 2nd hour of gallic acid induction (Fig. 4). This is in agreement with the observation that oxidative damage occurs in the gallic acid-induced cell death process^11,22^. Therefore, we tested whether gallic acid-induced cell death can be suppressed by antioxidants, such as captopril (CAP) and N-(2-mercaptopropionyl)-glycine (MPG). To answer this question, we preincubated HeLa cells with CAP (10 mM) or MPG (3 mM) for an hour and then co-treated the cells with gallic acid (50 μg/mL). The cells treated with CAP or MPG alone served as controls.

We found that treatment of CAP or MPG alone did not trigger cell death (Fig. 5Aiv–v, B). Importantly, CAP and MPG did suppress gallic acid-induced cell death (Fig. 5Aiv′–v′, B). Therefore, our data indicates that oxidative damage contribute to the execution of gallic acid-induced cell death process.

As mentioned above, our earlier study demonstrated that gallic acid triggered an iron-dependent cell death process, which can be suppressed by iron chelator deferoxamine (DFO, Fig. 1C)^22^. Here, we further validated our finding using a different iron chelator, ciclopirox (CPX). We found that cotreatment of CPX (5 µM) could inhibit gallic acid-induced cell death (Fig. 5Avi, vi′, B) in HeLa cells. It has been shown that depleting iron can inhibit the initiation of ferroptosis by suppressing lethal lipid peroxidation and imbalanced redox homeostasis^71,72^. Therefore, iron metabolism could serve as the upstream regulator of gallic acid-induced cell death pathway.

### Proposed molecular mechanism of gallic acid-induced cell death process

**Figure 6:**
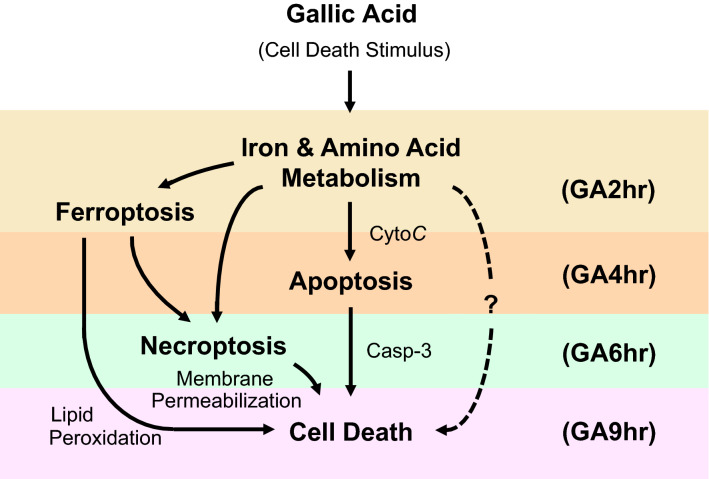
Proposed mechanisms of early (GA2hr), middle (GA4hr, GA6hr), and late (GA9hr) stages of gallic acid-induced cell death process. Dotted line indicates the unidentified cell death pathway(s) triggered by gallic acid.

Our RNA-sequencing study has identified the pathways enriched at the early (GA2hr), middle (GA4hr, GA6hr), and late (GA9hr) stages for contributing to the gallic acid-induced cell death process (Fig. 6).

At the early stage, the homeostatic iron regulator (HFE) was upregulated at the 2nd hour. This could be responsible to the iron-dependent cell death process activated by gallic acid. Besides, normal biosynthesis of amino acid was disrupted, leading to reduction of cellular GSH level. This could suppress the function of GPX4, resulting in defective lipid peroxide repair and lipid peroxidation. Moreover, the AMPK signaling pathway was enriched for triggering apoptotic pathway, and MAPK signaling pathway was enriched for triggering apoptotic and ferroptotic pathways.

At the middle stage, the lysosomal pathway was enriched at the 4th hour for triggering ferroptotic and necroptotic pathways. The TNF signaling pathway was enriched at the 6th hour for triggering necroptotic pathways. At the same time, the AMPK and MAPK signaling pathways continued to be enriched for activating apoptosis and ferroptosis in this time point.

At the late stage, cell death were identified by our enrichment analysis at the 9th hour, indicating that this is the end stage of the gallic acid-induced cell death process. Interestingly, the metabolic pathways were enriched at all stages of the cell death process. While the role of metabolism in cell death is unclear^30^, it could indirectly contribute indirectly to cell death execution through mTOR signaling pathway^69,70^, which was enriched at the late stage of gallic acid cell death process.

### Unanswered key questions

Key questions on the cellular response to gallic acid remain to be answered.

First, in consistent with our RNA-sequencing data that shows the enrichment of oxidation pathways in gallic acid-induced cell death process in HeLa cells, we and others have demonstrated that gallic acid can trigger cell death in human cancer cell lines with increase in the level of intracellular reactive oxygen species (ROS) and lipid peroxidation (ferroptotic feature)^11,22^. Interestingly, studies have also reported gallic acid with anti-oxidation property for cytoprotection in cellular and small animal models^17,74^. What cause the difference between these observations on gallic acid, ranging from promoting to inhibiting cell death, and from pro- to anti-oxidation?

The difference might not be due to the dosages of gallic acid, as the mentioned contradicting observations have been reported in the same dosages, ranging from 10 to 200 μg/mL (or mg/kg) in vitro and in vivo^11,17,74^ (and we applied 50 μg/mL gallic acid to HeLa cells in the present study). At the same time, it is possible that different cell types respond to the same dosages of gallic acid differently. For examples, the mentioned dosages often trigger cancer cells to undergo cell death and display increased in ROS level, while cardiac and neuronal cells often reveal reduction of ROS level and cytoprotection after administration of the same dosages of gallic acid in vitro and in vivo^11,17,74^.

Second, our RNA-sequencing data analysis reveals the enrichment of MAPK signaling pathway in the gallic acid-induced HeLa cells. This is supported by studies that demonstrated the activation of MAPK to promote cancer cell death by gallic acid^11^. However, downregulation of MAPK pathway by gallic acid has also been reported^75^. What cause the difference between these observations?

As mentioned above, gallic acid can trigger increase or decrease in ROS level, depending on cell types^11,17,22,74^. As ROS plays an important role in the activation of MAPK^76^, the different responses of the cells towards gallic acid could explain the different observations between activation and inhibition of MAPK pathways as reported in the other previous studies^11,75^.

Third, there is still downstream cell death pathway(s) triggered by gallic acid that remains to be identified. While targeting the upstream regulators and pathways of iron and amino acid metabolism by pharmacological approach can fully suppress gallic acid-induced cell death (Figs. 1C and 5)^22^, specific inhibitors targeting to the downstream regulators of apoptosis (Z-VAD-FMK, 50 µM), ferroptosis (ferrostatin-1, 2 µM; trolox, 50 µM; U0126, 10 µM), and necroptosis (necrostatin-1, 40 µM; necrosulfonamide, 5 µM), individually or all together, cannot suppress the gallic acid-induced cell death (Fig. S4)^22^. This indicates the existence of downstream cell death pathway(s) that has not yet been identified and targeted in the present study. However, these unrecognized downstream cell death pathways are mediated by the iron and amino acid metabolism, as targeting the iron and amino acid metabolism by pharmacological approach can fully suppress the gallic acid-induced cell death (Figs. 1C and 5).

How to identify the remaining downstream cell death pathway(s) that is triggered by gallic acid? Our present study only detected apoptotic, ferroptotic, and necroptotic features in the gallic acid-induced cell death^22^. However, we might miss other form(s) of possible cell death due to the lack of specific biochemical and molecular cell death markers for its detection and also the lack of targeting inhibitors for its manipulation^33^. Therefore, future discovery and further characterization of the existing and the new forms of cell death would allow the identification of those downstream cell death regulators mediated by gallic acid.

### Future perspective

How to identify the key regulators for determining the differential cellular responses of gallic acid? It could be possible that the cell models respond to gallic acid differently (e.g. promoting cell death versus cytoprotection^11,17^) display distinct differential gene expression profiles (molecular signatures). By comparing the molecular signatures between these cell models^11,17^, it will be able to identify the genes that display different expression in the models, which will suggest the potential key regulators that are responsible to the differential cellular responses of gallic acid.

Here, we have provided the first time-course RNA-sequencing data set of gallic acid-induced HeLa cell death model. Future studies that elucidate the gene expression profiles of the models with different cellular response toward gallic acid will enrich the pool of data sets. By revealing the changes in gene expression profiles in different cell types and conditions in the future studies, and comparing the data set generated in the present work, we will be in position to understand the molecular mechanisms governing the different cellular responses to gallic acid.

## Conclusions

We recently reported that gallic acid triggers an iron-dependent cell death mechanism with apoptotic, ferroptotic, and necroptotic features^22^. To reveal the molecular mechanisms that govern different stages of this cell death process, we now report the results of our time-course RNA-sequencing study. We found that the ferroptosis-related pathways are the first to be enriched (GA2hr), followed by the apoptosis-related pathways (GA4hr), and then the necroptosis-related pathways (GA6hr, GA9hr). We also demonstrated the importance of iron and amino acid metabolism for the initiation and execution of gallic acid-induced cell death. At the same time, we found that inhibiting the downstream pathways of apoptosis, ferroptosis, and necroptosis could not suppress the gallic acid-induced cell death, thereby indicating that gallic acid could trigger other cell death process(es) that remains to be identified. In summary, this study extends our understand of gallic acid-induced cell death process, laying a foundation for further investigation on the potential of using gallic acid to develop new anti-cancer therapies.

## Materials and methods

### Cell culture

The human cervical cancer HeLa cell line was purchased from the American Type Culture Collection (ATCC). The HeLa stable cell line expressing cytochrome *c* GFP was obtained from Douglas R. Green in the St. Jude Children's Research Hospital. The HeLa cells were cultured in DMEM/F-12 (Dulbecco’s Modified Eagle medium: Nutrient Mixture F-12) media, supplemented with 10% fetal bovine serum (FBS), 2 mM GlutaMAX, 100 U/mL penicillin, and 100 μg/mL streptomycin (Thermo Fisher Scientific, Carlsbad, CA, USA). The cells were maintained in optimal culture condition, at 37 degree Celsius (°C) under an atmosphere of 5% CO_2_. They were seeded on culture dishes for 24 h to achieve 50–60% confluence, before subjected to experiment.

### Cell death induction and inhibition

Gallic acid was purchased from Sigma-Aldrich (Burlington, MA, USA). It was diluted into the cell culture medium to achieve the working concentration of 50 µg/mL, immediately before being applied to the cells.

Ciclopirox (CPX), cysteine (CYS), deferoxamine (DFO), ferrostatin-1 (Fer-1), glutathione (GSH), N-(2-mercaptopropionyl)-glycine (MPG), necrostatin-1 (Nec-1), necrosulfonamide (NSA), trolox (Vit E), and U0126 were purchased from Sigma-Aldrich. Captopril (CAP) was purchased from Cell Signaling Technology (Danvers, MA, USA). Z-VAD-FMK was purchased from Enzo Life Sciences (Farmingdale, NY, USA). These agents were diluted to their working concentration (10 mM CAP, 5 µM CPX, 5 mM CYS, 200 µM DFO, 2 µM Fer-1, 10 mM GSH, 3 mM MPG, 40 µM Nec-1, 5 µM NSA, 50 µM Vit E, 10 µM U0126, and 50 µM Z-VAD-FMK) in the cell culture medium, before further treating the cells with or without gallic acid.

### Live cell staining for tracking nucleus, caspase-3 activation, plasma membrane permeability, and lipid peroxidation

To visualize nucleus, HeLa cells, cultured on a 35 mm glass-bottom dishes (MatTek Corporation, Ashland, MA, USA), were stained with 10 μg/mL of Hoechst 33342 blue nuclear dye (Thermo Fisher Scientific, Carlsbad, CA, USA) in the culture medium, for 20 min at 37 °C with 5% CO_2_. The stained cells were then washed twice with fresh medium and incubated for a further 10 min in order to remove the excessive dye, before the microscopy observations. Hoechst 33342 blue nuclear dye was observed with using DAPI channel (maximum excitation 405 nm, maximum emission 461 nm).

To detect caspase-3 activation, the NucView 530 Caspase-3 substrate (Biotium, Fremont, CA, USA), was diluted with cell culture medium 1:2000, and then applied to the cells 20 min before starting the live-cell imaging. The cells were incubated with the dye throughout the imaging experiment. The NucView® 530 Caspase-3 substrate Red was observed using Cy3 channel (maximum excitation 528 nm, maximum emission 563 nm).

To determine plasma membrane permeabilization, the IncuCyte Cytotox Red reagent (IncuCyte, Göttingen, Germany), which is impermeable to the plasma membrane, was diluted with cell culture medium 1:1000, and then applied to the cells 20 min before starting the live-cell imaging. This reagent can be also incubated with the cells throughout the entire experiments of the time-lapse live-cell imaging. The IncuCyte® Cytotox Red reagent was observed using Cy5 channel (maximum excitation 612 nm, maximum emission 631 nm).

To detect lipid peroxidation, the sensor reagent from the Image-iT™ Lipid Peroxidation Kit (Thermo Fisher Scientific, Carlsbad, CA, USA) was diluted with cell culture medium 1:1000, and incubated the cells for 30 min. The cells were then washed twice with fresh cell culture medium immediately before being imaged by confocal microscope.

### Time-lapse live-cell confocal microscopy

The inverted confocal microscope with environmental control (37 °C, 5% CO_2_) was used to perform time-lapse live-cell imaging. To begin, the microscope was first prewarmed to 37 °C with environmental control and stage heater at least 1 h before imaging. This can allow all of the microscope components to reach thermo-equilibrium, in order to avoid the drift of focus and shift of x–y plane due to the thermal expansion and contraction of the components. Then, a 35 mm MatTek glass bottom dish of cultured cells was placed on the stage of the inverted microscope, and capture cell images with the scanner of LSM780 (Carl Zeiss, Jena, Germany). Cell images were obtained using a 40 × Plan-Apochromat objective with N.A. 1.4. Cells were incubated in optimal culture condition, at 37 °C with 5% CO_2_ throughout the live-cell imaging process. The cell images were analyzed using Zen and AxioVision software (Carl Zeiss).

### RNA isolation

The human cervical cancer HeLa cells were plated onto Corning tissue culture dishes (100 mm, Corning, NY) with seeding density 1 × 10^6^, and were cultured in the optimal condition as described above for 1 day to reach 80% cell confluency, before being subjected to experiment. To induce cell death, the HeLa cells were exposure to 50 µg/mL gallic acid in culture medium, and were then cultured for 2 h, 4 h, 6 h, and 9 h. The untreated cells served as control (Ctrl). Three biological replicates were performed at each time point mentioned above.

The total RNA in these corresponding conditions was harvested using TRIzol Reagent (Thermo Fisher Scientific). In brief, the culture medium of the cultured cells at each of the timepoint was removed from the culture dish. Then, 2 mL of TRIzol was added on the dish, and was mixed thoroughly with the cells by pipetting. The well cell-TRIzol mixture was then transferred to the corresponding individual Seal-Rite 2.0 ml microcentrifuge tube (Seal-Rite, USA Scientific, Ocala, FL, USA), and was stored in − 80 °C. The total RNA was isolated and purified using the Direct-zol™ RNA MiniPrep (Zymo Research, Irvine, CA, USA) with the standard protocol as described by the manufacturer.

### RNA quantification and qualification

The RNA integrity was measured with the standard protocols as described by the following manufacturers. The RNA purity was determined according to the using the NanoPhotometer spectrophotometer (IMPLEN, CA, USA). The RNA concentration was measured using the NanoDrop spectrophotometer, and also Qubit RNA Assay Kit in Qubit 2.0 fluorometer (Life Technologies, CA, USA). The RNA integrity was checked using the RNA Nano 600 Assay Kit and the Bioanalyzer 2100 system (Agilent Technologies, CA, USA).

### Library construction and sequencing

The RNA was first subjected to the Ribosomal RNA depletion, which was performed with Ribo-zero Magnetic Gold Kit (Illumina Inc., San Diego, CA, USA). Samples were randomly primed and fragmented based on manufacturer’s recommendation (NEBNext® Ultra™ II Directional RNA Library Prep Kit, Illumina). The first strand was synthesized with the Protoscript II Reverse Transcriptase with a longer extension period (30 min for 42 °C). All remaining steps for library construction were performed according to the NEBNext® Ultra™ II Directional RNA Library Prep Kit for Illumina. The Illumina 8-nt dual-indices were used for multiplexing. The constructed library samples were pooled and sequenced on Illumina HiSeq 4000 sequencer for 150 bp read length in paired-end mode. RNA-Seq reads were aligned to Homo sapiens (human) reference genome GRCh38 (hg38). Bowtie2 v2.2.8 software was used to build index of reference genome, and paired-end clean reads of RNA-Seq were aligned to the reference genome using HISAT2 v2.0.4^77^. HISAT2 was run with ‘–rna-strandness RF’. DEG analysis was done using Cuffdiff.

### Reverse transcription polymerase chain reaction (RT-PCR)

SuperScript IV reverse transcriptase system (Thermo Fisher Scientific, Carlsbad, CA, USA) was used to perform reverse transcription. Polymerase chain reaction (PCR) was performed using Taq DNA polymerase with ThermoPol buffer (New England BioLabs, Ipswich, MA, USA), with initial denaturation at 95 °C for 30 s, followed by 30 cycles of denaturation at 95 °C for 30 s, annealing at 55 °C for 30 s, and extension at 68 °C for 10 s. Final extension was performed at 68 °C for 5 min. Electrophoresis was performed using 1.5% agarose gel.

### Bioinformatics analysis

The Principal Components Analysis (PCA) three-dimensional plot (PCA3D) was created using the top 3 PCs to illustrate the relationship among samples. The FPKM (Fragments Per Kilobase of gene model per Million reads per sample) for each gene of each sample was used to calculate the contribution of each Principal Component, using prcomp function in R. The PCA3D plot was then generated using scatterplot3d package in R. The heat map of unsupervised hierarchical clustering, Venn diagram, Gene Ontology (GO) and Kyoto Encyclopedia of Genes and Genomes (KEGG) analyses were performed by Novogene (Beijing, China) as the standard analysis service.

### Cell count and statistical analysis

Three biological replicates were performed for each experiment, with at least 100 cells were counted for each replicate. One-way analysis of variance (ANOVA), followed by Tukey's test for post-hoc analysis, were performed for statistical comparison. Mean ± standard deviation (s.d.) is shown in the result. Differences are considered to be significant when the *p*-value is < 0.05.

## Supplementary Information

Supplementary Information 1.

Supplementary Information 2.

Supplementary Video 1.

## Data Availability

The raw and processed RNA-sequencing data are posted in the Gene Expression Omnibus (GEO) database (GSE158788), and is available for public access.

## Abbreviations

Cyto*C*: Cytochrome *c*
DIC: Differential interference contrast
GA: Gallic acid
GO: Gene Ontology
KEGG: Kyoto Encyclopedia of Genes and Genomes
PCA: Principal component analysis
