# Supplementary material for: Gene expression profile analysis of gallic acid-induced cell death process
Source: Sci Rep. 2021 Aug 18;11:16743. doi: 10.1038/s41598-021-96174-1 (PMC8373985; doi:10.1038/s41598-021-96174-1)
Supplement: Supplementary file 1 — Supplementary Information 1. [file 41598_2021_96174_MOESM1_ESM.pdf]

Figure S1. Loss of plasma membrane integrity shortly after caspse-3 activation in gallic acid-induced cell death process. Time-lapse live-cell confocal microscopy of the same group of cytochrome *c*-GFP expressing HeLa cells after treatment with 50 μg/mL of gallic acid for 6th to 9th hour. (***i-iv***) Top row, merged images of differential interference contrast (DIC) microscopy, cytochrome *c*-GFP, and nucleus; (***i'-iv'***) Middle row, DIC and caspase red substrate; (***i''-iv''***); Bottom row, DIC and plasma membrane-permeable dye. Arrows: blue, nuclear condensation; green, cytochrome *c* release from mitochondria to cytosol; pink, plasma membrane permeabilization; red, caspase-3 activation. Scale bar: 10 μm. Corresponding images of the same group of cells at different timepoints are available in Figure 1A and Supplementary Video 1.

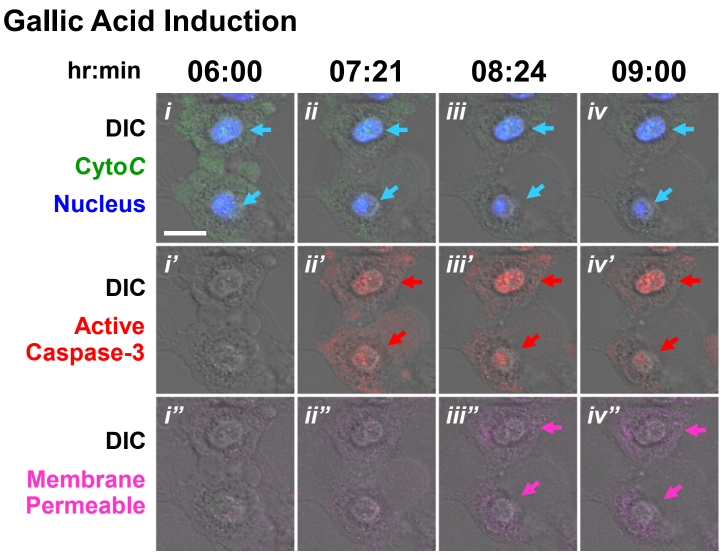

Figure S2. Suppression of gallic acid-induced cell death by iron chelator deferoxamine. Representing phase images of HeLa cells *(i)* cultured in normal condition (Untreated), *(ii)* treated with deferoxamine (DFO, 200 μM) for 24 hours, *(iii)* treated with gallic acid (GA, 50 μg/mL) for 24 hours, and *(iv)* co-treated with gallic acid and deferoxamine (GA + DFO) for 24 hours. Scale bar: 100 μm.

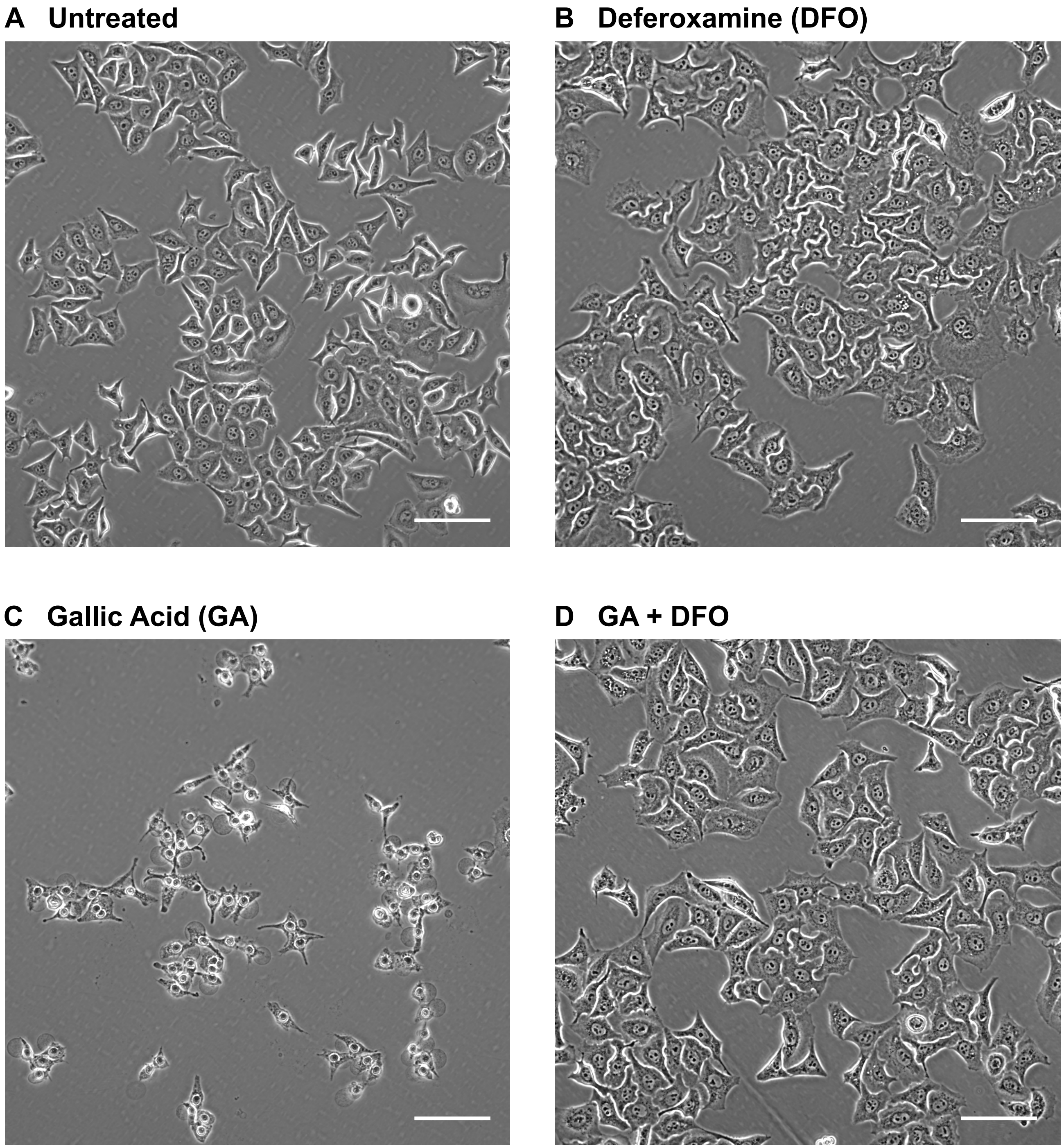

Figure S3. Change of gene expression in gallic acid-induced human cancer cells detected by RT-PCR gel analysis. mRNA levels of AKAP1, BTBD3, PPT1, RPL13AP3, UBC, and ZNF254 in human cervical cancer HeLa cells, human melanoma A-375 cells, human osteosarcoma MG-63 cells, and human bladder carcinoma 5637 cells, with (GA) or without (Ctrl) treated with 50 μg/mL gallic acid for 9 hours. RPL13AP3 and UBC served as housekeeping genes. List of primer sequences is available in Table S2.

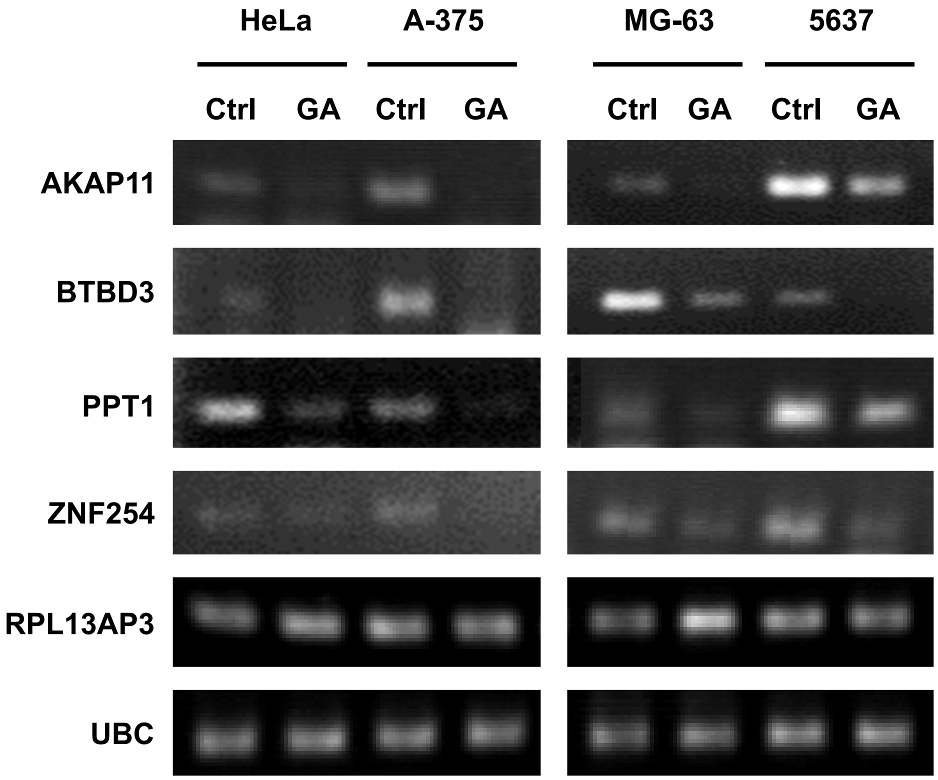

Figure S4. Incapability of inhibiting downstream regulators of apoptosis, ferroptosis, and necroptosis for suppressing gallic acid-induced cell death. Quantification of the cell death events, including nuclear condensation (blue) and plasma membrane rupture (grey), in HeLa cells treated with ***(i)*** medium alone (Control), ***(ii)*** deferoxamine (DFO, 200 μM), ***(iii)*** ferrostatin-1 (Fer-1, 2 μM), ***(iv)*** trolox (Vit E, 50 μM), ***(v)*** U0126 (10 μM), ***(vi)*** Fer-1, Vit E and U0126, ***(vii)*** necrostatin-1 (Nec-1, 40 μM), ***(viii)*** necrosulfonamide (NSA, 5 μM), ***(ix)*** Nec-1 and NSA, ***(x)*** Z-VAD-FMK (50 μM), ***(xi)*** combined ~~iii to x~~, with ***(i'–xi')*** or without ***(i–xi)*** co-treatment of gallic acid (50 μg/mL) for 24 hours. Mean ± s.d.; n = 3. The data was evaluated using one-way analysis of variance (ANOVA), followed by Tukey's test for post-hoc analysis. * $p < 0.01$.

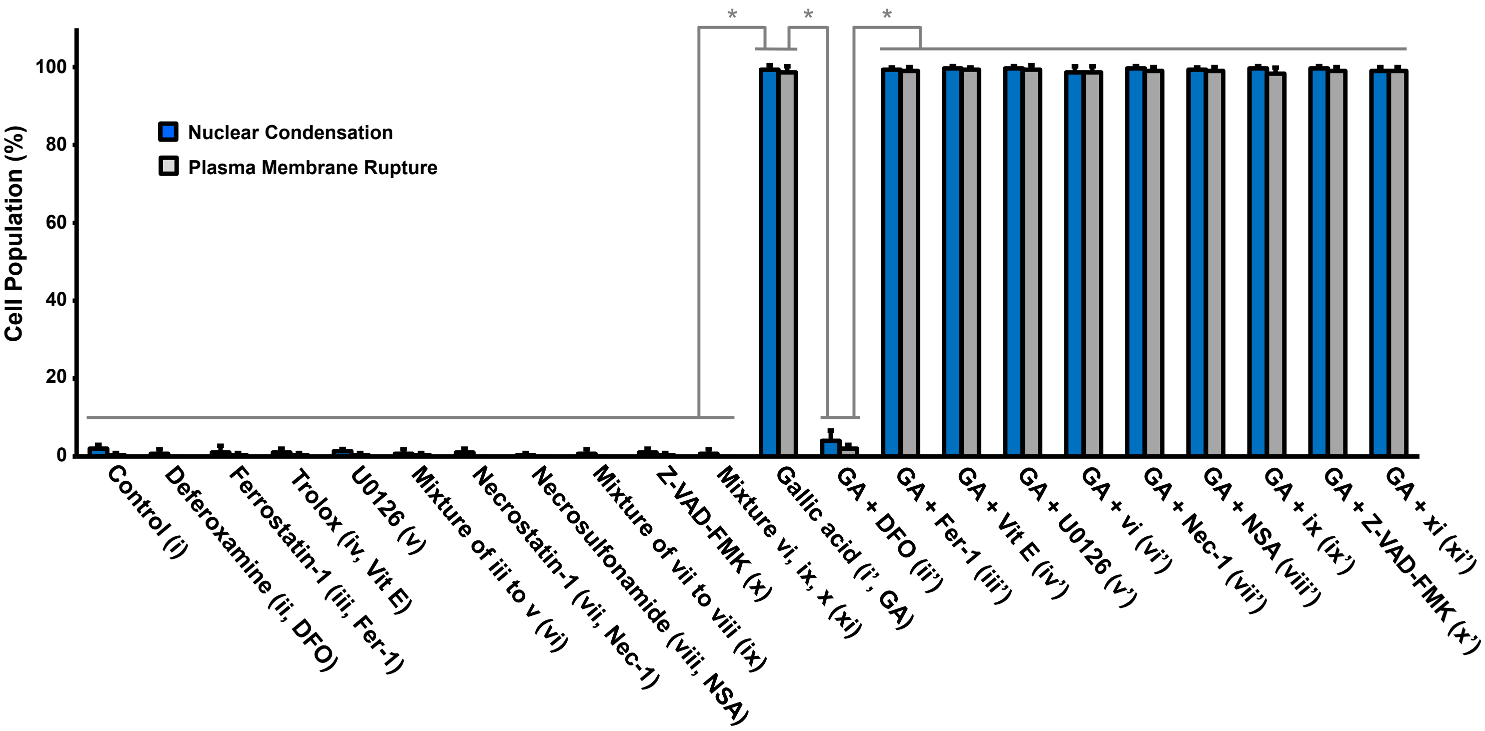

Table S1. Summary of RNA-sequencing data set.
A list summarizes the sample names, and the corresponding read depth, data quality, and overall alignment rate.

Sample	Number of read pairs	Number of bases (G)	Q20	Q30	Overall alignment rate
GA0hr_a	71330940	10.77	97.01%	93.25%	80.42%
GA0hr_b	71029178	10.73	97.07%	93.33%	80.38%
GA0hr_c	68281480	10.31	97.21%	93.56%	82.27%
GA2hr_a	62212698	9.39	97.02%	93.23%	80.78%
GA2hr_b	64339334	9.72	96.89%	93.16%	79.05%
GA2hr_c	67574478	10.20	96.92%	93.01%	85.29%
GA4hr_a	61030986	9.22	97.06%	93.32%	81.07%
GA4hr_b	64310240	9.71	97.14%	93.39%	80.34%
GA4hr_c	69649370	10.52	97.02%	93.17%	79.62%
GA6hr_a	74759662	11.29	96.71%	92.87%	76.22%
GA6hr_b	69309164	10.47	96.68%	92.92%	76.10%
GA6hr_c	63214050	9.55	96.81%	92.98%	76.40%
GA9hr_a	65524154	9.89	96.76%	92.96%	72.95%
GA9hr_b	57755714	8.72	96.38%	92.28%	75.98%
GA9hr_c	57775330	8.72	96.56%	92.64%	71.71%

Table S2. **List of primers for RT-PCR.**

Gene	Accession number	Forward primer	Reverse primer	Amplicon
AKAP1	NM_003488.4	GAAGTCCTTCTGGACAG TGTGATG	GAATCAGAGGAAGACCAGTTGGACTG	145
BTBD3	NM_014962.4	GTCGAACCTGCTGCTTTTCTCGC	CACAGGCTCTGGCAAGGTGAGGG	133
PPT1	NM_000310.4	GTACTCCAAAGTTGTTCAGGAAC	GCCAAGAAGATGCTGTGGTTGCG	96
RPL13AP3	NR_004844.1	GAAAAAGCGGATGGTGGTTCCTGC	GTGGCTGTCACTGCCTGGTACTTC	123
UBC	BC039193.1	GAGCCCAGTGACACCATCGAGAATG	GAGCACCAGGTGCAGGGTGGACTC	162
ZNF254	NM_001278677.2	GAGGAGTGGCAACACCTGGACATTG	CCTTGTTCCAGACAGGTGATCAG	131

Table S3. **List of top 80 up-regulated genes at 2nd hour (GA2hr) of gallic acid induction, compared with the control cells (Ctrl, GA0hr).**

Sort Order	Gene Symbol	Definition	Accession	log_2 fold change GA2hr vs. Ctrl
1	EEF1D	eukaryotic translation elongation factor 1 delta	ENSG00000104529	19.06824821
2	MEGF8	multiple EGF like domains 8	ENSG00000105429	18.36854625
3	CNOT7	CCR4-NOT transcription complex subunit 7	ENSG00000198791	18.06396642
4	ANAPC11	anaphase promoting complex subunit 11	ENSG00000141552	17.69839777
5	IGF1R	insulin like growth factor 1 receptor	ENSG00000140443	16.9471791
6	CAPNS1	calpain small subunit 1	ENSG00000126247	16.78501673
7	IBTK	inhibitor of Bruton tyrosine kinase	ENSG00000005700	16.44619483
8	SNX12	sorting nexin 12	ENSG00000147164	15.56424044
9	MAGEA6	MAGE family member A6	ENSG00000197172	15.36876018
10	RFFL	ring finger and FYVE like domain containing E3 ubiquitin protein ligase	ENSG00000092871	15.31068822
11	TSC22D3	TSC22 domain family member 3	ENSG00000157514	14.93739484
12	NBPF26	NBPF member 26	ENSG00000273136	14.5719896
13	TMBIM6	transmembrane BAX inhibitor motif containing 6	ENSG00000139644	14.48174678
14	TRIM36	tripartite motif containing 36	ENSG00000152503	14.45364584
15	IST1	IST1, ESCRT-III associated factor	ENSG00000182149	13.41112067
16	ST6GALNAC6	ST6 N-acetylgalactosaminide alpha-2,6-sialyltransferase 6	ENSG00000160408	13.4059738
17	WASF1	WAS protein family member 1	ENSG00000112290	13.38654874
18	NAA80	N(alpha)-acetyltransferase 80, NatH catalytic subunit	ENSG00000243477	13.20611838
19	DDX1	DEAD-box helicase 1	ENSG00000079785	13.15592502
20	CPSF7	cleavage and polyadenylation specific factor 7	ENSG00000149532	12.67755219
21	GNAL	G protein subunit alpha L	ENSG00000141404	12.50352763
22	ENOPH1	enolase-phosphatase 1	ENSG00000145293	12.47786472
23	NUBPL	nucleotide binding protein like	ENSG00000151413	12.46619484
24	MOB3C	MOB kinase activator 3C	ENSG00000142961	12.33295962
25	NAV3	neuron navigator 3	ENSG00000067798	12.27404446
26	NOSTRIN	nitric oxide synthase trafficking	ENSG00000163072	12.11087182
27	TNFAIP3	TNF alpha induced protein 3	ENSG00000118503	11.89880985
28	ZC3H12C	zinc finger CCCH-type containing 12C	ENSG00000149289	11.80400279
29	ZFYVE27	zinc finger FYVE-type containing 27	ENSG00000155256	11.53911013
30	AC008878.1	novel transcript	ENSG00000267952	11.37870426
31	AMBRA1	autophagy and beclin 1 regulator 1	ENSG00000110497	11.34675971
32	ZC3HC1	zinc finger C3HC-type containing 1	ENSG00000091732	11.257893
33	TAF6	TATA-box binding protein associated factor 6	ENSG00000106290	11.24817444
34	RBFOX2	RNA binding fox-1 homolog 2	ENSG00000100320	11.13143761
35	CRYBG2	crystallin beta-gamma domain containing 2	ENSG00000176092	11.10914991
36	AASDH	aminoadipate-semialdehyde dehydrogenase	ENSG00000157426	11.0380135
37	ICOSLG	inducible T cell costimulator ligand	ENSG00000160223	11.03600185
38	METTL8	methyltransferase like 8	ENSG00000123600	10.93431653
39	ZNF83	zinc finger protein 83	ENSG00000167766	10.90831747
40	TMUB1	transmembrane and ubiquitin like domain containing 1	ENSG00000164897	10.90603842
41	GOLGA3	golgin A3	ENSG00000090615	10.84461228
42	TMEM205	transmembrane protein 205	ENSG00000105518	10.82097669
43	RABL2B	RAB, member of RAS oncogene family like 2B	ENSG00000079974	10.76431338
44	PFKFB3	6-phosphofructo-2-kinase/fructose-2,6-biphosphatase 3	ENSG00000170525	10.50598564
45	FKRP	fukutin related protein	ENSG00000181027	10.47126191
46	NPIPB13	nuclear pore complex interacting protein family, member B13	ENSG00000198064	10.3980036
47	MEIS3	Meis homeobox 3	ENSG00000105419	10.37392153
48	C16orf58	chromosome 16 open reading frame 58	ENSG00000140688	10.2515372
49	SUPT5H	SPT5 homolog, DSIF elongation factor subunit	ENSG00000196235	10.24758123
50	GBGT1	globoside alpha-1,3-N-acetylgalactosaminyltransferase 1 (FORS blood group)	ENSG00000148288	10.23302043
51	TMEM170A	transmembrane protein 170A	ENSG00000166822	10.12710719
52	SELENOF	selenoprotein F	ENSG00000183291	10.10440903
53	TBC1D15	TBC1 domain family member 15	ENSG00000121749	10.04293488
54	GJC1	gap junction protein gamma 1	ENSG00000182963	10.03603048
55	VIPR1	vasoactive intestinal peptide receptor 1	ENSG00000114812	10.01385571
56	BMP2K	BMP2 inducible kinase	ENSG00000138756	9.903949175
57	ZNF264	zinc finger protein 264	ENSG00000083844	9.896909976
58	RNPS1	RNA binding protein with serine rich domain 1	ENSG00000205937	9.783124059
59	PRDM10	PR/SET domain 10	ENSG00000170325	9.779997348
60	NARF	nuclear prelamin A recognition factor	ENSG00000141562	9.774534415
61	ARNTL	aryl hydrocarbon receptor nuclear translocator like	ENSG00000133794	9.718189273
62	LIPT1	lipoyltransferase 1	ENSG00000144182	9.69518474
63	BANK1	B cell scaffold protein with ankyrin repeats 1	ENSG00000153064	9.669147382
64	IP6K2	inositol hexakisphosphate kinase 2	ENSG00000068745	9.66433917
65	TSC22D3	TSC22 domain family member 3	ENSG00000157514	9.658122838
66	BDNF	brain derived neurotrophic factor	ENSG00000176697	9.617138956
67	LDAH	lipid droplet associated hydrolase	ENSG00000118961	9.552308859
68	NLRP1	NLR family pyrin domain containing 1	ENSG00000091592	9.508896473
69	FANCM	FA complementation group M	ENSG00000187790	9.473554033
70	AMN1	antagonist of mitotic exit network 1 homolog	ENSG00000151743	9.437954884
71	ERGIC3	ERGIC and golgi 3	ENSG00000125991	9.432066568
72	PCK2	phosphoenolpyruvate carboxykinase 2, mitochondrial	ENSG00000100889	9.334570535
73	STARD10	StAR related lipid transfer domain containing 10	ENSG00000214530	9.330577091
74	ORC4	origin recognition complex subunit 4	ENSG00000115947	9.328016049
75	ZNF771	zinc finger protein 771	ENSG00000179965	9.318552723
76	ARNTL	aryl hydrocarbon receptor nuclear translocator like	ENSG00000133794	9.298534888
77	PPP1R18	protein phosphatase 1 regulatory subunit 18	ENSG00000146112	9.190756093
78	FAM110A	family with sequence similarity 110 member A	ENSG00000125898	9.185888146
79	DNM3	dynamin 3	ENSG00000197959	9.167088966
80	PRR13	proline rich 13	ENSG00000205352	9.112532875

Table S4. **List of top 80 down-regulated genes at 2nd hour (GA2hr) of gallic acid induction, compared with the control cells (Ctrl, GA0hr).**

Sort Order	Gene Symbol	Definition	Accession	log_2 fold change GA2hr vs. Ctrl
1	GART	phosphoribosylglycinamide formyltransferase	ENSG00000159131	-22.3894913
2	PRMT1	protein arginine methyltransferase 1	ENSG00000126457	-19.14362071
3	RAP1B	RAP1B, member of RAS oncogene family	ENSG00000127314	-18.59345313
4	FBXW11	F-box and WD repeat domain containing 11	ENSG00000072803	-17.53866638
5	HBP1	HMG-box transcription factor 1	ENSG00000105856	-17.50233458
6	CASK	calcium/calmodulin dependent serine protein kinase	ENSG00000147044	-17.07090708
7	EEF1D	eukaryotic translation elongation factor 1 delta	ENSG00000104529	-16.70719734
8	PDLIM5	PDZ and LIM domain 5	ENSG00000163110	-16.5288589
9	CHST8	carbohydrate sulfotransferase 8	ENSG00000124302	-16.25225888
10	RACGAP1	Rac GTPase activating protein 1	ENSG00000161800	-15.83544422
11	KMT2C	lysine methyltransferase 2C	ENSG00000055609	-15.79392636
12	UBA52	ubiquitin A-52 residue ribosomal protein fusion product 1	ENSG00000221983	-14.52691682
13	ZMYND8	zinc finger MYND-type containing 8	ENSG00000101040	-14.48927097
14	BIRC2	baculoviral IAP repeat containing 2	ENSG00000110330	-14.44506805
15	XRCC4	X-ray repair cross complementing 4	ENSG00000152422	-14.39167352
16	ZFP62	ZFP62 zinc finger protein	ENSG00000196670	-13.97808815
17	MXRA7	matrix remodeling associated 7	ENSG00000182534	-13.8042115
18	EPB41	erythrocyte membrane protein band 4.1	ENSG00000159023	-13.7143129
19	LIMA1	LIM domain and actin binding 1	ENSG00000050405	-12.92930748
20	USP19	ubiquitin specific peptidase 19	ENSG00000172046	-12.83810779
21	HRH1	histamine receptor H1	ENSG00000196639	-12.78391486
22	FNBP1	formin binding protein 1	ENSG00000187239	-12.5187586
23	RPS6KC1	ribosomal protein S6 kinase C1	ENSG00000136643	-12.25728726
24	TMEM106C	transmembrane protein 106C	ENSG00000134291	-12.14646172
25	ME2	malic enzyme 2	ENSG00000082212	-12.10896454
26	B3GALT6	beta-1,3-galactosyltransferase 6	ENSG00000176022	-12.0511821
27	RACGAP1	Rac GTPase activating protein 1	ENSG00000161800	-12.04871796
28	PTPRJ	protein tyrosine phosphatase, receptor type J	ENSG00000149177	-11.98819327
29	TCF7L2	transcription factor 7 like 2	ENSG00000148737	-11.75562778
30	DEPDC5	DEP domain containing 5	ENSG00000100150	-11.65360394
31	SMARCE1	SWI/SNF related, matrix associated, actin dependent regulator of chromatin	ENSG00000073584	-11.63260275
32	TMEM250	transmembrane protein 250	ENSG00000238227	-11.59540348
33	ATP6V0E2	ATPase H+ transporting V0 subunit e2	ENSG00000171130	-11.53185963
34	IMPDH1	inosine monophosphate dehydrogenase 1	ENSG00000106348	-11.51630072
35	LPP	LIM domain containing preferred translocation partner in lipoma	ENSG00000145012	-11.43788028
36	SMARCA4	SWI/SNF related, matrix associated, actin dependent regulator of chromatin	ENSG00000127616	-11.21862263
37	DNAJB6	DnaJ heat shock protein family (Hsp40) member B6	ENSG00000105993	-11.08476205
38	EMSY	EMSY, BRCA2 interacting transcriptional repressor	ENSG00000158636	-11.04535488
39	DIP2C	disco interacting protein 2 homolog C	ENSG00000151240	-10.87962556
40	EPB41	erythrocyte membrane protein band 4.1	ENSG00000159023	-10.78144932
41	RAB30	RAB30, member RAS oncogene family	ENSG00000137502	-10.72850215
42	USP2	ubiquitin specific peptidase 2	ENSG00000036672	-10.72649548
43	PRPSAP1	phosphoribosyl pyrophosphate synthetase associated protein 1	ENSG00000161542	-10.69766529
44	TAX1BP1	Tax1 binding protein 1	ENSG00000106052	-10.66295756
45	URGCP-MRPS24	URGCP-MRPS24 readthrough	ENSG00000270617	-10.61604177
46	RTN2	reticulon 2	ENSG00000125744	-10.61097791
47	DAPK1	death associated protein kinase 1	ENSG00000196730	-10.59675169
48	DNM1	dynamin 1	ENSG00000106976	-10.46823505
49	DGCR8	DGCR8, microprocessor complex subunit	ENSG00000128191	-10.42441017
50	KDSR	3-ketodihydrosphingosine reductase	ENSG00000119537	-10.32745316
51	CYLD	CYLD lysine 63 deubiquitinase	ENSG00000083799	-10.26040189
52	CERS6	ceramide synthase 6	ENSG00000172292	-10.23294643
53	DDX50	DExD-box helicase 50	ENSG00000107625	-10.22391088
54	ALG11	ALG11, alpha-1,2-mannosyltransferase	ENSG00000253710	-10.19392956
55	COPS7B	COP9 signalosome subunit 7B	ENSG00000144524	-10.18760982
56	TRIM45	tripartite motif containing 45	ENSG00000134253	-10.1640194
57	ABHD11	abhydrolase domain containing 11	ENSG00000106077	-10.12011882
58	BTBD3	BTB domain containing 3	ENSG00000132640	-10.05957126
59	WASHC2C	WASH complex subunit 2C	ENSG00000172661	-9.995783444
60	TPM3	tropomyosin 3	ENSG00000143549	-9.894572329
61	E2F7	E2F transcription factor 7	ENSG00000165891	-9.884998206
62	ZNF23	zinc finger protein 23	ENSG00000167377	-9.883114941
63	ZNF84	zinc finger protein 84	ENSG00000198040	-9.869733166
64	SEC23B	Sec23 homolog B, coat complex II component	ENSG00000101310	-9.856401768
65	ODR4	odr-4 GPCR localization factor homolog	ENSG00000157181	-9.770669872
66	HSPA8	heat shock protein family A (Hsp70) member 8	ENSG00000109971	-9.731396306
67	ABCA2	ATP binding cassette subfamily A member 2	ENSG00000107331	-9.681351373
68	SMARCA4	SWI/SNF related, matrix associated, actin dependent regulator of chromatin	ENSG00000127616	-9.666795061
69	TRAF5	TNF receptor associated factor 5	ENSG00000082512	-9.661599791
70	HSD17B4	hydroxysteroid 17-beta dehydrogenase 4	ENSG00000133835	-9.616125441
71	ZNF205	zinc finger protein 205	ENSG00000122386	-9.611024797
72	ZNFX1	zinc finger NFX1-type containing 1	ENSG00000124201	-9.584579402
73	RELCH	RAB11 binding and LisH domain, coiled-coil and HEAT repeat containing	ENSG00000134444	-9.579717503
74	PACRGL	parkin coregulated like	ENSG00000163138	-9.546092861
75	MATR3	matrin 3	ENSG00000015479	-9.539149167
76	EBPL	EBP like	ENSG00000123179	-9.524189733
77	PTER	phosphotriesterase related	ENSG00000165983	-9.495855027
78	KIAA1324L	KIAA1324 like	ENSG00000164659	-9.460079033
79	ZNF510	zinc finger protein 510	ENSG00000081386	-9.441053292
80	TEAD2	TEA domain transcription factor 2 [Source:HGNC Symbol;Acc:HGNC:11715]	ENSG00000074219	-9.436584079

Table S5. List of top 80 up-regulated genes at 4th hour (GA4hr) of gallic acid induction, compared with the control cells (Ctrl, GA0hr).

Sort Order	Gene Symbol	Definition	Accession	log_2 fold change GA4hr vs. Ctrl
1	MEGF8	multiple EGF like domains 8	ENSG00000105429	18.52017488
2	IGF1R	insulin like growth factor 1 receptor	ENSG00000140443	16.48819269
3	CNOT7	CCR4-NOT transcription complex subunit 7	ENSG00000198791	16.44482348
4	SNX12	sorting nexin 12 [Source:HGNC Symbol;Acc:HGNC:14976]	ENSG00000147164	15.75914635
5	CAPNS1	calpain small subunit 1	ENSG00000126247	15.12294665
6	TSC22D3	TSC22 domain family member 3	ENSG00000157514	14.48185468
7	CPSF7	cleavage and polyadenylation specific factor 7	ENSG00000149532	14.15478433
8	TNFAIP3	TNF alpha induced protein 3	ENSG00000118503	12.9517683
9	DDX1	DEAD-box helicase 1	ENSG00000079785	12.48448727
10	SLAIN1	SLAIN motif family member 1	ENSG00000139737	12.45576256
11	ZC3H12C	zinc finger CCCH-type containing 12C	ENSG00000149289	12.38856698
12	HMOX2	heme oxygenase 2	ENSG00000103415	12.38464767
13	RBFOX2	RNA binding fox-1 homolog 2	ENSG00000100320	12.30126008
14	KPNB1	karyopherin subunit beta 1	ENSG00000108424	12.26000902
15	USP19	ubiquitin specific peptidase 19	ENSG00000172046	12.12895185
16	TMEM170A	transmembrane protein 170A	ENSG00000166822	11.7279881
17	AMBRA1	autophagy and beclin 1 regulator 1	ENSG00000110497	11.43662111
18	TSC22D3	TSC22 domain family member 3	ENSG00000157514	11.39182249
19	CREM	cAMP responsive element modulator	ENSG00000095794	11.34315902
20	NUBPL	nucleotide binding protein like	ENSG00000151413	11.24493716
21	ZFYVE27	zinc finger FYVE-type containing 27	ENSG00000155256	11.15512408
22	METTL8	methyltransferase like 8	ENSG00000123600	11.13410594
23	TMUB1	transmembrane and ubiquitin like domain containing 1	ENSG00000164897	11.03777189
24	WDR81	WD repeat domain 81	ENSG00000167716	11.03486107
25	ERGIC3	ERGIC and golgi 3	ENSG00000125991	10.8264783
26	NOSTRIN	nitric oxide synthase trafficking	ENSG00000163072	10.79409236
27	TAF6	TATA-box binding protein associated factor 6	ENSG00000106290	10.55228801
28	GBGT1	globoside alpha-1,3-N-acetylgalactosaminyltransferase 1	ENSG00000148288	10.53897235
29	PPP3CA	protein phosphatase 3 catalytic subunit alpha	ENSG00000138814	10.48596317
30	RABL2B	RAB, member of RAS oncogene family like 2B	ENSG00000079974	10.46500063
31	EPB41	erythrocyte membrane protein band 4.1	ENSG00000159023	10.43481341
32	RAD51	RAD51 recombinase	ENSG00000051180	10.3683031
33	SPHK2	sphingosine kinase 2	ENSG00000063176	10.1985397
34	GOLGA3	golgin A3	ENSG00000090615	10.15434449
35	ORC4	origin recognition complex subunit 4	ENSG00000115947	10.13274433
36	AC008878.1	novel transcript	ENSG00000267952	10.11288293
37	PRDM10	PR/SET domain 10	ENSG00000170325	10.02159568
38	AP1G1	adaptor related protein complex 1 subunit gamma 1	ENSG00000166747	9.98106478
39	ZC3HC1	zinc finger C3HC-type containing 1	ENSG00000091732	9.926212064
40	KLHL13	kelch like family member 13	ENSG00000003096	9.84166037
41	RNPS1	RNA binding protein with serine rich domain 1	ENSG00000205937	9.820864047
42	POP1	POP1 homolog, ribonuclease P/MRP subunit	ENSG00000104356	9.733829025
43	ATG13	autophagy related 13	ENSG00000175224	9.621872666
44	NIF3L1	NGG1 interacting factor 3 like 1	ENSG00000196290	9.545916489
45	DNM3	dynamin 3	ENSG00000197959	9.544993506
46	WARS	tryptophanyl-tRNA synthetase	ENSG00000140105	9.450182812
47	IST1	IST1, ESCRT-III associated factor	ENSG00000182149	9.374117627
48	KDM3A	lysine demethylase 3A	ENSG00000115548	9.313928426
49	TINF2	TERF1 interacting nuclear factor 2	ENSG00000092330	9.273985619
50	MAP3K6	mitogen-activated protein kinase kinase kinase 6	ENSG00000142733	9.273180543
51	AP1M1	adaptor related protein complex 1 subunit mu 1	ENSG00000072958	9.271501084
52	UQCC1	ubiquinol-cytochrome c reductase complex assembly factor 1	ENSG00000101019	9.261261261
53	SLC4A1AP	solute carrier family 4 member 1 adaptor protein	ENSG00000163798	9.234137386
54	MEIS3	Meis homeobox 3	ENSG00000105419	9.223524005
55	CXorf56	chromosome X open reading frame 56	ENSG00000018610	9.164814219
56	ZNF83	zinc finger protein 83	ENSG00000167766	9.089116761
57	FKBP10	FK506 binding protein 10	ENSG00000141756	9.063080612
58	GJC1	gap junction protein gamma 1	ENSG00000182963	8.961630563
59	RABL2B	RAB, member of RAS oncogene family like 2B	ENSG00000079974	8.934791709
60	NVL	nuclear VCP-like	ENSG00000143748	8.85887415
61	AP3D1	adaptor related protein complex 3 subunit delta 1	ENSG00000065000	8.852921767
62	HIKESHI	Hikeshi, heat shock protein nuclear import factor	ENSG00000149196	8.847119712
63	PPP1R18	protein phosphatase 1 regulatory subunit 18	ENSG00000146112	8.824667349
64	M6PR	mannose-6-phosphate receptor, cation dependent	ENSG00000003056	8.707802928
65	CIRBP	cold inducible RNA binding protein	ENSG00000099622	8.694870165
66	KIF23	kinesin family member 23	ENSG00000137807	8.652801686
67	ZNF211	zinc finger protein 211	ENSG00000121417	8.648057158
68	BDNF	brain derived neurotrophic factor	ENSG00000176697	8.637074223
69	PFKM	phosphofructokinase, muscle	ENSG00000152556	8.603985482
70	ZNF771	zinc finger protein 771	ENSG00000179965	8.564005899
71	IFRD2	interferon related developmental regulator 2	ENSG00000214706	8.558309207
72	ADGRB2	adhesion G protein-coupled receptor B2	ENSG00000121753	8.552501845
73	FOLR1	folate receptor 1	ENSG00000110195	8.526962078
74	NDUFA3	NADH:ubiquinone oxidoreductase subunit A3	ENSG00000170906	8.482859079
75	ATP2B1	ATPase plasma membrane Ca2+ transporting 1	ENSG00000070961	8.478763055
76	DPP9	dipeptidyl peptidase 9	ENSG00000142002	8.47119141
77	TMEM129	transmembrane protein 129	ENSG00000168936	8.470637653
78	MAP2K4	mitogen-activated protein kinase kinase 4	ENSG00000065559	8.454710721
79	PRR13	proline rich 13	ENSG00000205352	8.444206307
80	CALCOCO2	calcium binding and coiled-coil domain 2	ENSG00000136436	8.37542808

Table S6. **List of top 80 down-regulated genes at 4th hour (GA4hr) of gallic acid induction, compared with the control cells (Ctrl, GA0hr).**

Sort Order	Gene Symbol	Definition	Accession	log_2 fold change GA4hr vs. Ctrl
1	ASXL1	ASXL transcriptional regulator 1	ENSG00000171456	-20.37764055
2	CDK7	cyclin dependent kinase 7	ENSG00000134058	-18.34982977
3	SYBU	syntabulin	ENSG00000147642	-15.74584499
4	MXRA7	matrix remodeling associated 7	ENSG00000182534	-14.76114278
5	HOXC10	homeobox C10	ENSG00000180818	-14.73953002
6	KAT6A	lysine acetyltransferase 6A	ENSG00000083168	-14.68119182
7	IFNGR1	interferon gamma receptor 1	ENSG00000027697	-14.35482411
8	WDR25	WD repeat domain 25	ENSG00000176473	-14.15740238
9	DCAF4	DDB1 and CUL4 associated factor 4	ENSG00000119599	-13.54739378
10	SEC23B	Sec23 homolog B, coat complex II component	ENSG00000101310	-11.99258434
11	TMEM106C	transmembrane protein 106C	ENSG00000134291	-11.99220887
12	ME2	malic enzyme 2	ENSG00000082212	-11.8499259
13	NEIL1	nei like DNA glycosylase 1	ENSG00000140398	-11.82193528
14	NFXL1	nuclear transcription factor, X-box binding like 1	ENSG00000170448	-11.70372031
15	C9orf72	chromosome 9 open reading frame 72	ENSG00000147894	-11.54192697
16	ZNF195	zinc finger protein 195	ENSG00000005801	-11.42777101
17	MZF1	myeloid zinc finger 1	ENSG00000099326	-11.38707363
18	B3GALT6	beta-1,3-galactosyltransferase 6	ENSG00000176022	-11.3020013
19	SLC2A8	solute carrier family 2 member 8	ENSG00000136856	-10.98394695
20	LCLAT1	lysocardiolipin acyltransferase 1	ENSG00000172954	-10.87948606
21	RNPS1	RNA binding protein with serine rich domain 1	ENSG00000205937	-10.85459125
22	TEAD2	TEA domain transcription factor 2	ENSG00000074219	-10.55944083
23	MSRB3	methionine sulfoxide reductase B3	ENSG00000174099	-10.52009302
24	CERS6	ceramide synthase 6	ENSG00000172292	-10.4086078
25	ODR4	odr-4 GPCR localization factor homolog	ENSG00000157181	-10.37379527
26	KAT6A	lysine acetyltransferase 6A	ENSG00000083168	-10.32740671
27	NAA60	N(alpha)-acetyltransferase 60, NatF catalytic subunit	ENSG00000122390	-10.29394548
28	UBB	ubiquitin B	ENSG00000170315	-10.28967784
29	VWA5A	von Willebrand factor A domain containing 5A	ENSG00000110002	-10.22500523
30	SPTAN1	spectrin alpha, non-erythrocytic 1	ENSG00000197694	-10.20361935
31	CCSAP	centriole, cilia and spindle associated protein	ENSG00000154429	-10.177758
32	ABI1	abl interactor 1	ENSG00000136754	-10.10600669
33	AC002310.4	novel protein	ENSG00000260869	-10.04287185
34	KDSR	3-ketodihydrosphingosine reductase	ENSG00000119537	-10.00310498
35	CLN5	CLN5, intracellular trafficking protein	ENSG00000102805	-9.988761256
36	USP45	ubiquitin specific peptidase 45	ENSG00000123552	-9.986742426
37	CTNND1	catenin delta 1	ENSG00000198561	-9.968762778
38	TM7SF3	transmembrane 7 superfamily member 3	ENSG00000064115	-9.94192079
39	ZNF431	zinc finger protein 431	ENSG00000196705	-9.92062353
40	MED25	mediator complex subunit 25	ENSG00000104973	-9.853547452
41	POU2F1	POU class 2 homeobox 1	ENSG00000143190	-9.767578534
42	BTBD3	BTB domain containing 3	ENSG00000132640	-9.719259491
43	TAF11L4	TATA-box binding protein associated factor 11 like 4	ENSG00000284283	-9.670333739
44	TRAIP	TRAF interacting protein	ENSG00000183763	-9.62181744
45	ZNF827	zinc finger protein 827	ENSG00000151612	-9.534186634
46	PLS3	plastin 3	ENSG00000102024	-9.45367118
47	TTPAL	alpha tocopherol transfer protein like	ENSG00000124120	-9.411161762
48	SMARCE1	SWI/SNF related, matrix associated, actin dependent regulator of chromatin	ENSG00000073584	-9.378281097
49	AGAP3	ArfGAP with GTPase domain, ankyrin repeat and PH domain 3	ENSG00000133612	-9.284872461
50	ACAD10	acyl-CoA dehydrogenase family member 10	ENSG00000111271	-9.216174569
51	USP21	ubiquitin specific peptidase 21	ENSG00000143258	-9.19789061
52	PYCARD	PYD and CARD domain containing	ENSG00000103490	-9.191422148
53	HTT	huntingtin	ENSG00000197386	-9.128993088
54	ABCA5	ATP binding cassette subfamily A member 5	ENSG00000154265	-9.11415609
55	ZNF329	zinc finger protein 329	ENSG00000181894	-9.106276499
56	DOCK7	dedicator of cytokinesis 7	ENSG00000116641	-9.043274149
57	DGCR8	DGCR8, microprocessor complex subunit	ENSG00000128191	-9.020094388
58	ZNF821	zinc finger protein 821	ENSG00000102984	-9.009704479
59	ZNF41	zinc finger protein 41	ENSG00000147124	-8.993067426
60	MON2	MON2 homolog, regulator of endosome-to-Golgi trafficking	ENSG00000061987	-8.988977082
61	DIP2C	disco interacting protein 2 homolog C	ENSG00000151240	-8.985494197
62	ENO2	enolase 2	ENSG00000111674	-8.976898783
63	NRDC	nardilysin convertase	ENSG00000078618	-8.967905624
64	NF1	neurofibromin 1	ENSG00000196712	-8.957894452
65	SLC25A23	solute carrier family 25 member 23	ENSG00000125648	-8.836302942
66	BARD1	BRCA1 associated RING domain 1	ENSG00000138376	-8.835400884
67	PPT1	palmitoyl-protein thioesterase 1	ENSG00000131238	-8.821668421
68	AMMECR1L	AMMECR1 like	ENSG00000144233	-8.779063262
69	BRCC3	BRCA1/BRCA2-containing complex subunit 3	ENSG00000185515	-8.755531002
70	SCRN3	secernin 3	ENSG00000144306	-8.741723436
71	CBS	cystathionine-beta-synthase	ENSG00000160200	-8.708433591
72	CXXC5	CXXC finger protein 5	ENSG00000171604	-8.690214735
73	PMP22	peripheral myelin protein 22	ENSG00000109099	-8.667890612
74	CACFD1	calcium channel flower domain containing 1	ENSG00000160325	-8.647050793
75	TNFAIP8L1	TNF alpha induced protein 8 like 1	ENSG00000185361	-8.631603156
76	MB	myoglobin	ENSG00000198125	-8.553694874
77	PRDM15	PR/SET domain 15	ENSG00000141956	-8.541010348
78	NDUFAF6	NADH:ubiquinone oxidoreductase complex assembly factor 6	ENSG00000156170	-8.534318799
79	MAP4K4	mitogen-activated protein kinase kinase kinase kinase 4	ENSG00000071054	-8.522265128
80	DEPDC5	DEP domain containing 5	ENSG00000100150	-8.486889174

Table S7. List of top 80 up-regulated genes at 6th hour (GA6hr) of gallic acid induction, compared with the control cells (Ctrl, GA0hr).

Sort Order	Gene Symbol	Definition	Accession	log_2 fold change GA6hr vs. Ctrl
1	EEF1D	eukaryotic translation elongation factor 1 delta	ENSG00000104529	20.7281929
2	MEGF8	multiple EGF like domains 8	ENSG00000105429	16.30752102
3	GNAL	G protein subunit alpha L	ENSG00000141404	15.97440563
4	PRDM5	PR/SET domain 5	ENSG00000138738	14.65931306
5	UTRN	utrophin	ENSG00000152818	14.37179177
6	NAA80	N(alpha)-acetyltransferase 80, NatH catalytic subunit	ENSG00000243477	13.40300642
7	TNFAIP3	TNF alpha induced protein 3	ENSG00000118503	13.37393599
8	LDAH	lipid droplet associated hydrolase	ENSG00000118961	13.35163806
9	PTPRK	protein tyrosine phosphatase, receptor type K	ENSG00000152894	13.13956507
10	DDX1	DEAD-box helicase 1	ENSG00000079785	13.1238789
11	SLC12A2	solute carrier family 12 member 2	ENSG00000064651	13.03249357
12	DPP9	dipeptidyl peptidase 9	ENSG00000142002	12.92428661
13	KLHL13	kelch like family member 13	ENSG00000003096	12.72929817
14	ZNF714	zinc finger protein 714	ENSG00000160352	12.3799821
15	GOLGA3	golgin A3	ENSG00000090615	12.21107061
16	HMOX2	heme oxygenase 2	ENSG00000103415	12.09251202
17	PCK2	phosphoenolpyruvate carboxykinase 2, mitochondrial	ENSG00000100889	11.99142508
18	SLAIN1	SLAIN motif family member 1	ENSG00000139737	11.7765482
19	STARD10	StAR related lipid transfer domain containing 10	ENSG00000214530	11.46468002
20	ZFYVE27	zinc finger FYVE-type containing 27	ENSG00000155256	11.37805773
21	TSC22D3	TSC22 domain family member 3	ENSG00000157514	11.27933891
22	NUBPL	nucleotide binding protein like	ENSG00000151413	11.2130174
23	RBFOX2	RNA binding fox-1 homolog 2	ENSG00000100320	11.09743103
24	SPHK2	sphingosine kinase 2	ENSG00000063176	11.01862285
25	ZC3H12C	zinc finger CCCH-type containing 12C	ENSG00000149289	10.88731804
26	SUPT5H	SPT5 homolog, DSIF elongation factor subunit	ENSG00000196235	10.88262246
27	CAMSAP2	calmodulin regulated spectrin associated protein family member 2	ENSG00000118200	10.83370267
28	TAF6	TATA-box binding protein associated factor 6	ENSG00000106290	10.32512453
29	AC008878.1	novel transcript	ENSG00000267952	10.20524238
30	PRDM10	PR/SET domain 10	ENSG00000170325	10.14824331
31	BANK1	B cell scaffold protein with ankyrin repeats 1	ENSG00000153064	10.04880957
32	GJC1	gap junction protein gamma 1	ENSG00000182963	10.02212214
33	PPP1R18	protein phosphatase 1 regulatory subunit 18	ENSG00000146112	10.01345809
34	RABL2B	RAB, member of RAS oncogene family like 2B	ENSG00000079974	9.992882312
35	ANKRD11	ankyrin repeat domain 11	ENSG00000167522	9.877322492
36	NPIPB13	nuclear pore complex interacting protein family, member B13	ENSG00000198064	9.856793503
37	LRRC37B	leucine rich repeat containing 37B	ENSG00000185158	9.823619345
38	ORC4	origin recognition complex subunit 4	ENSG00000115947	9.752984771
39	METTL8	methyltransferase like 8	ENSG00000123600	9.67347388
40	PFKFB3	6-phosphofructo-2-kinase/fructose-2,6-biphosphatase 3	ENSG00000170525	9.662583163
41	DIAPH1	diaphanous related formin 1	ENSG00000131504	9.661826269
42	TMUB1	transmembrane and ubiquitin like domain containing 1	ENSG00000164897	9.651933617
43	AKAP13	A-kinase anchoring protein 13	ENSG00000170776	9.624450856
44	ARNTL	aryl hydrocarbon receptor nuclear translocator like	ENSG00000133794	9.613585647
45	AP1M1	adaptor related protein complex 1 subunit mu 1	ENSG00000072958	9.506226467
46	PFKFB3	6-phosphofructo-2-kinase/fructose-2,6-biphosphatase 3	ENSG00000170525	9.416025339
47	IKZF2	IKAROS family zinc finger 2	ENSG00000030419	9.408199126
48	LIPT1	lipoyltransferase 1	ENSG00000144182	9.377780111
49	GBGT1	globoside alpha-1,3-N-acetylgalactosaminyltransferase 1 (FORS blood group)	ENSG00000148288	9.313488416
50	NDUFA3	NADH:ubiquinone oxidoreductase subunit A3	ENSG00000170906	9.288137395
51	UQCC1	ubiquinol-cytochrome c reductase complex assembly factor 1	ENSG00000101019	9.25283864
52	KDM3A	lysine demethylase 3A	ENSG00000115548	9.242027272
53	USP3	ubiquitin specific peptidase 3	ENSG00000140455	9.23620034
54	CAMK2G	calcium/calmodulin dependent protein kinase II gamma	ENSG00000148660	9.138609912
55	ATXN2	ataxin 2	ENSG00000204842	9.110386484
56	NIF3L1	NGG1 interacting factor 3 like 1	ENSG00000196290	9.085469087
57	TRAPPC9	trafficking protein particle complex 9	ENSG00000167632	9.07489226
58	STAU2	staufen double-stranded RNA binding protein 2	ENSG00000040341	9.062955725
59	MEIS3	Meis homeobox 3	ENSG00000105419	9.058825438
60	PIAS2	protein inhibitor of activated STAT 2	ENSG00000078043	9.053102651
61	UBXN6	UBX domain protein 6	ENSG00000167671	9.02946848
62	PFKM	phosphofructokinase, muscle	ENSG00000152556	9.020904299
63	SGCE	sarcoglycan epsilon	ENSG00000127990	9.012167981
64	USP19	ubiquitin specific peptidase 19	ENSG00000172046	9.000036615
65	FKRP	fukutin related protein	ENSG00000181027	8.964345063
66	VIPR1	vasoactive intestinal peptide receptor 1	ENSG00000114812	8.956780662
67	WARS	tryptophanyl-tRNA synthetase	ENSG00000140105	8.904441784
68	COCH	cochlin	ENSG00000100473	8.900892262
69	CYB5R2	cytochrome b5 reductase 2	ENSG00000166394	8.897330884
70	POP1	POP1 homolog, ribonuclease P/MRP subunit	ENSG00000104356	8.815093929
71	ANAPC11	anaphase promoting complex subunit 11	ENSG00000141552	8.801495193
72	ARPC4	actin related protein 2/3 complex subunit 4	ENSG00000241553	8.791221108
73	CTDSPL2	CTD small phosphatase like 2	ENSG00000137770	8.755439442
74	COMMD9	COMM domain containing 9	ENSG00000110442	8.743350726
75	XPNPEP1	X-prolyl aminopeptidase 1	ENSG00000108039	8.710648906
76	FLRT3	fibronectin leucine rich transmembrane protein 3	ENSG00000125848	8.650745693
77	PRUNE2	prune homolog 2	ENSG00000106772	8.560644773
78	ANAPC11	anaphase promoting complex subunit 11	ENSG00000141552	8.510764168
79	RPL5	ribosomal protein L5	ENSG00000122406	8.408327101
80	TMEM170A	transmembrane protein 170A	ENSG00000166822	8.375747664

Table S8. **List of top 80 down-regulated genes at 6th hour (GA6hr) of gallic acid induction, compared with the control cells (Ctrl, GA0hr).**

Sort Order	Gene Symbol	Definition	Accession	log_2 fold change GA6hr vs. Ctrl
1	SLC9B2	solute carrier family 9 member B2	ENSG00000164038	-21.00332643
2	PRMT1	protein arginine methyltransferase 1	ENSG00000126457	-20.72858321
3	C21orf58	chromosome 21 open reading frame 58	ENSG00000160298	-16.53575075
4	IMPDH1	inosine monophosphate dehydrogenase 1	ENSG00000106348	-14.8162887
5	ZFP30	ZFP30 zinc finger protein	ENSG00000120784	-14.31802268
6	LCLAT1	lysocardiolipin acyltransferase 1	ENSG00000172954	-14.29057329
7	LIMA1	LIM domain and actin binding 1	ENSG00000050405	-13.16933315
8	TEX264	testis expressed 264	ENSG00000164081	-13.02792706
9	RPL32	ribosomal protein L32	ENSG00000144713	-12.61823836
10	WNK2	WNK lysine deficient protein kinase 2	ENSG00000165238	-12.23837855
11	TMEM250	transmembrane protein 250	ENSG00000238227	-12.16947767
12	CLIP1	CAP-Gly domain containing linker protein 1	ENSG00000130779	-12.13542521
13	WASHC2C	WASH complex subunit 2C	ENSG00000172661	-12.04944941
14	IL17RC	interleukin 17 receptor C	ENSG00000163702	-11.82550917
15	DHCR24	24-dehydrocholesterol reductase	ENSG00000116133	-11.75825129
16	TMEM106C	transmembrane protein 106C	ENSG00000134291	-11.73359075
17	STAG1	stromal antigen 1	ENSG00000118007	-11.64063762
18	CASP7	caspase 7	ENSG00000165806	-11.4918531
19	ITM2C	integral membrane protein 2C	ENSG00000135916	-11.47001862
20	ADGRG2	adhesion G protein-coupled receptor G2	ENSG00000173698	-11.05210762
21	CAPN1	calpain 1	ENSG00000014216	-10.94880985
22	TMEM218	transmembrane protein 218	ENSG00000150433	-10.87629857
23	MXRA7	matrix remodeling associated 7	ENSG00000182534	-10.79875707
24	PRKCH	protein kinase C eta	ENSG00000027075	-10.73911139
25	ABCA2	ATP binding cassette subfamily A member 2	ENSG00000107331	-10.70210993
26	SMARCE1	SWI/SNF related, matrix associated, actin dependent regulator of chromatin	ENSG00000073584	-10.58076382
27	TRIQK	triple QxxK/R motif containing	ENSG00000205133	-10.487297
28	PTPRJ	protein tyrosine phosphatase, receptor type J	ENSG00000149177	-10.46757878
29	DGCR8	DGCR8, microprocessor complex subunit	ENSG00000128191	-10.45489078
30	RAVER2	ribonucleoprotein, PTB binding 2	ENSG00000162437	-10.42318469
31	NFATC4	nuclear factor of activated T cells 4	ENSG00000100968	-10.24869951
32	CASP7	caspase 7	ENSG00000165806	-10.1646701
33	B4GALT4	beta-1,4-galactosyltransferase 4	ENSG00000121578	-10.10507116
34	GTPBP10	GTP binding protein 10	ENSG00000105793	-10.07084137
35	ZNF468	zinc finger protein 468	ENSG00000204604	-10.02683784
36	DIAPH2	diaphanous related formin 2	ENSG00000147202	-9.931285115
37	AC002310.4	novel protein	ENSG00000260869	-9.906085189
38	BTBD3	BTB domain containing 3	ENSG00000132640	-9.830861009
39	ATP6V0E2	ATPase H+ transporting V0 subunit e2	ENSG00000171130	-9.797688238
40	PLCXD1	phosphatidylinositol specific phospholipase C X domain containing 1	ENSG00000182378	-9.779612119
41	AQP1	aquaporin 1 (Colton blood group)	ENSG00000240583	-9.779223472
42	CNOT6L	CCR4-NOT transcription complex subunit 6 like	ENSG00000138767	-9.766363435
43	GYS1	glycogen synthase 1	ENSG00000104812	-9.742979378
44	FAM92A	family with sequence similarity 92 member A	ENSG00000188343	-9.741225957
45	PPP2R3C	protein phosphatase 2 regulatory subunit B"gamma	ENSG00000092020	-9.693277126
46	USP32	ubiquitin specific peptidase 32	ENSG00000170832	-9.673753932
47	SLC35E2B	solute carrier family 35 member E2B	ENSG00000189339	-9.670747481
48	SLC12A8	solute carrier family 12 member 8	ENSG00000221955	-9.655699594
49	GEMIN7	gem nuclear organelle associated protein 7	ENSG00000142252	-9.557362379
50	PMP22	peripheral myelin protein 22	ENSG00000109099	-9.4912213
51	EEF1D	eukaryotic translation elongation factor 1 delta	ENSG00000104529	-9.482506077
52	KDSR	3-ketodihydrosphingosine reductase	ENSG00000119537	-9.468180154
53	SDSL	serine dehydratase like	ENSG00000139410	-9.449368655
54	HSP90AA1	heat shock protein 90 alpha family class A member 1	ENSG00000080824	-9.435286547
55	ANKRD42	ankyrin repeat domain 42	ENSG00000137494	-9.39911062
56	HIST2H2BF	histone cluster 2 H2B family member f	ENSG00000203814	-9.359603881
57	CYP20A1	cytochrome P450 family 20 subfamily A member 1	ENSG00000119004	-9.336773745
58	ZNF71	zinc finger protein 71	ENSG00000197951	-9.300856409
59	DDX47	DEAD-box helicase 47	ENSG00000213782	-9.286271866
60	NDUFB1	NADH:ubiquinone oxidoreductase subunit B1	ENSG00000183648	-9.196965318
61	MSL3	MSL complex subunit 3	ENSG00000005302	-9.144844454
62	SYNJ2	synaptojanin 2	ENSG00000078269	-9.129830814
63	PRKACB	protein kinase cAMP-activated catalytic subunit beta	ENSG00000142875	-9.092700582
64	HSD17B4	hydroxysteroid 17-beta dehydrogenase 4	ENSG00000133835	-8.970010733
65	UBE2J2	ubiquitin conjugating enzyme E2 J2	ENSG00000160087	-8.909678584
66	LIG4	DNA ligase 4	ENSG00000174405	-8.755519638
67	CEP57L1	centrosomal protein 57 like 1	ENSG00000183137	-8.742394
68	TBC1D2B	TBC1 domain family member 2B	ENSG00000167202	-8.738436748
69	IL1R1	interleukin 1 receptor type 1	ENSG00000115594	-8.714619111
70	PCCA	propionyl-CoA carboxylase subunit alpha	ENSG00000175198	-8.643745896
71	AFMID	arylformamidase	ENSG00000183077	-8.6230427
72	TK2	thymidine kinase 2, mitochondrial	ENSG00000166548	-8.607330314
73	CACFD1	calcium channel flower domain containing 1	ENSG00000160325	-8.595073143
74	YBEY	ybeY metalloendoribonuclease	ENSG00000182362	-8.537444617
75	DBN1	drebrin 1	ENSG00000113758	-8.529838067
76	ANKRD11	ankyrin repeat domain 11	ENSG00000167522	-8.52438706
77	TRMT10A	tRNA methyltransferase 10A	ENSG00000145331	-8.51832323
78	ZNF329	zinc finger protein 329	ENSG00000181894	-8.518098719
79	FAM13C	family with sequence similarity 13 member C	ENSG00000148541	-8.471229344
80	SPATS2L	spermatogenesis associated serine rich 2 like	ENSG00000196141	-8.447877283

Table S9. List of top 80 up-regulated genes at 9th hour (GA9hr) of gallic acid induction, compared with the control cells (Ctrl, GA0hr).

Sort Order	Gene Symbol	Definition	Accession	log_2 fold change GA9hr vs. Ctrl
1	GNAL	G protein subunit alpha L	ENSG00000141404	17.11617742
2	IGF1R	insulin like growth factor 1 receptor	ENSG00000140443	16.86283053
3	UTRN	utrophin	ENSG00000152818	16.08871004
4	RFFL	ring finger and FYVE like domain containing E3 ubiquitin protein ligase	ENSG00000092871	15.37393567
5	MEGF8	multiple EGF like domains 8	ENSG00000105429	15.23859133
6	DDX1	DEAD-box helicase 1	ENSG00000079785	15.12765853
7	PRDM5	PR/SET domain 5	ENSG00000138738	14.38932884
8	NAA80	N(alpha)-acetyltransferase 80, NatH catalytic subunit	ENSG00000243477	14.31474753
9	TNFAIP3	TNF alpha induced protein 3	ENSG00000118503	13.9920498
10	TSC22D3	TSC22 domain family member 3	ENSG00000157514	13.8499826
11	TMBIM6	transmembrane BAX inhibitor motif containing 6	ENSG00000139644	13.30761946
12	ZC3H12C	zinc finger CCCH-type containing 12C	ENSG00000149289	12.75911402
13	NUBPL	nucleotide binding protein like	ENSG00000151413	12.619917
14	ZNF714	zinc finger protein 714	ENSG00000160352	12.59568657
15	KLHL13	kelch like family member 13	ENSG00000003096	12.55416182
16	IST1	IST1, ESCRT-III associated factor	ENSG00000182149	12.45021987
17	MOB3C	MOB kinase activator 3C	ENSG00000142961	12.40758644
18	SLC12A2	solute carrier family 12 member 2	ENSG00000064651	12.30131078
19	USP19	ubiquitin specific peptidase 19	ENSG00000172046	12.19782822
20	TMEM170A	transmembrane protein 170A	ENSG00000166822	11.96895158
21	AMBRA1	autophagy and beclin 1 regulator 1	ENSG00000110497	11.88131626
22	CPSF7	cleavage and polyadenylation specific factor 7	ENSG00000149532	11.85687128
23	NOSTRIN	nitric oxide synthase trafficking	ENSG00000163072	11.79560752
24	TAF6	TATA-box binding protein associated factor 6	ENSG00000106290	11.7890046
25	SEC22A	SEC22 homolog A, vesicle trafficking protein	ENSG00000121542	11.65163198
26	ZKSCAN1	zinc finger with KRAB and SCAN domains 1	ENSG00000106261	11.57319552
27	BDH1	3-hydroxybutyrate dehydrogenase 1	ENSG00000161267	11.49664188
28	SLAIN1	SLAIN motif family member 1	ENSG00000139737	11.48581078
29	PCK2	phosphoenolpyruvate carboxykinase 2, mitochondrial	ENSG00000100889	11.35125676
30	TSC22D3	TSC22 domain family member 3	ENSG00000157514	11.28679941
31	RABL2B	RAB, member of RAS oncogene family like 2B	ENSG00000079974	11.1551871
32	BANK1	B cell scaffold protein with ankyrin repeats 1	ENSG00000153064	11.11038372
33	SPHK2	sphingosine kinase 2	ENSG00000063176	10.86423704
34	NDUFA3	NADH:ubiquinone oxidoreductase subunit A3	ENSG00000170906	10.85181571
35	SMAD4	SMAD family member 4	ENSG00000141646	10.67283568
36	PRDM10	PR/SET domain 10	ENSG00000170325	10.50029735
37	ANAPC11	anaphase promoting complex subunit 11	ENSG00000141552	10.4314976
38	VIPR1	vasoactive intestinal peptide receptor 1	ENSG00000114812	10.40519054
39	KDM3A	lysine demethylase 3A	ENSG00000115548	10.36711336
40	ZC3HC1	zinc finger C3HC-type containing 1	ENSG00000091732	10.3055116
41	LDAH	lipid droplet associated hydrolase	ENSG00000118961	10.12936352
42	SELENOF	selenoprotein F	ENSG00000183291	10.03295524
43	TIMM50	translocase of inner mitochondrial membrane 50	ENSG00000105197	9.823774502
44	OSBPL6	oxysterol binding protein like 6	ENSG00000079156	9.821955446
45	COQ8B	coenzyme Q8B	ENSG00000123815	9.796041554
46	EPB41	erythrocyte membrane protein band 4.1	ENSG00000159023	9.748147533
47	GJC1	gap junction protein gamma 1	ENSG00000182963	9.72470924
48	ZNF264	zinc finger protein 264	ENSG00000083844	9.619867092
49	RGS20	regulator of G protein signaling 20	ENSG00000147509	9.558571762
50	NKAPD1	NKAP domain containing 1	ENSG00000150776	9.556083591
51	TMBIM6	transmembrane BAX inhibitor motif containing 6	ENSG00000139644	9.55464865
52	PLEKHG4	pleckstrin homology and RhoGEF domain containing G4	ENSG00000196155	9.529547042
53	TRIM36	tripartite motif containing 36	ENSG00000152503	9.517545401
54	RPS6KC1	ribosomal protein S6 kinase C1	ENSG00000136643	9.471610323
55	CTDSPL2	CTD small phosphatase like 2	ENSG00000137770	9.455682087
56	ADGRB2	adhesion G protein-coupled receptor B2	ENSG00000121753	9.428944938
57	MEIS3	Meis homeobox 3	ENSG00000105419	9.368529759
58	JMJD4	jumonji domain containing 4	ENSG00000081692	9.358800354
59	ZNF573	zinc finger protein 573	ENSG00000189144	9.35557201
60	MAP3K6	mitogen-activated protein kinase kinase kinase 6	ENSG00000142733	9.344110057
61	TMUB1	transmembrane and ubiquitin like domain containing 1	ENSG00000164897	9.199647727
62	PFKFB3	6-phosphofructo-2-kinase/fructose-2,6-biphosphatase 3	ENSG00000170525	9.197169273
63	GBGT1	globoside alpha-1,3-N-acetylgalactosaminyltransferase 1 (FORS blood group)	ENSG00000148288	9.196120858
64	ZNF211	zinc finger protein 211	ENSG00000121417	9.185391635
65	SLC35A3	solute carrier family 35 member A3	ENSG00000117620	9.119948882
66	IFT27	intraflagellar transport 27	ENSG00000100360	9.076705962
67	METTL8	methyltransferase like 8	ENSG00000123600	9.076054311
68	SLC4A1AP	solute carrier family 4 member 1 adaptor protein	ENSG00000163798	9.049887043
69	DNM3	dynamin 3	ENSG00000197959	8.963629493
70	DEPDC5	DEP domain containing 5	ENSG00000100150	8.961153045
71	CCDC117	coiled-coil domain containing 117	ENSG00000159873	8.927328647
72	CYB5R2	cytochrome b5 reductase 2	ENSG00000166394	8.922827032
73	C2orf88	chromosome 2 open reading frame 88	ENSG00000187699	8.89956227
74	TMEM129	transmembrane protein 129	ENSG00000168936	8.881759875
75	PTRH1	peptidyl-tRNA hydrolase 1 homolog	ENSG00000187024	8.783310214
76	MYO1B	myosin IB	ENSG00000128641	8.749631344
77	HMGB1	high mobility group box 1	ENSG00000189403	8.702173242
78	HADHB	hydroxyacyl-CoA dehydrogenase trifunctional multienzyme complex subunit beta	ENSG00000138029	8.688940554
79	AUTS2	AUTS2, activator of transcription and developmental regulator	ENSG00000158321	8.679183088
80	KIF23	kinesin family member 23	ENSG00000137807	8.675563097

Table S10. List of top 80 down-regulated genes at 9th hour (GA9hr) of gallic acid induction, compared with the control cells (Ctrl, GA0hr).

Sort Order	Gene Symbol	Definition	Accession	log_2 fold change GA9hr vs. Ctrl
1	TACC3	transforming acidic coiled-coil containing protein 3	ENSG00000013810	-19.75754627
2	KAT6B	lysine acetyltransferase 6B	ENSG00000156650	-18.44204445
3	PITPNB	phosphatidylinositol transfer protein beta	ENSG00000180957	-17.75142311
4	RAP1B	RAP1B, member of RAS oncogene family	ENSG00000127314	-17.59345313
5	B2M	beta-2-microglobulin	ENSG00000166710	-17.31263024
6	PDLIM5	PDZ and LIM domain 5	ENSG00000163110	-16.39196516
7	GGA1	golgi associated, gamma adaptin ear containing, ARF binding protein 1	ENSG00000100083	-16.39078193
8	ATM	ATM serine/threonine kinase	ENSG00000149311	-15.76871602
9	DST	dystonin	ENSG00000151914	-14.34429145
10	WASF1	WAS protein family member 1	ENSG00000112290	-14.28332228
11	HMBS	hydroxymethylbilane synthase	ENSG00000256269	-14.25202598
12	CAST	calpastatin	ENSG00000153113	-14.14167153
13	SYPL1	synaptophysin like 1	ENSG00000008282	-14.13304714
14	MBD1	methyl-CpG binding domain protein 1	ENSG00000141644	-14.08467346
15	ZNF254	zinc finger protein 254	ENSG00000213096	-14.02066091
16	SUMF2	sulfatase modifying factor 2	ENSG00000129103	-13.15360243
17	B3GALT6	beta-1,3-galactosyltransferase 6	ENSG00000176022	-13.11431708
18	PTPRF	protein tyrosine phosphatase, receptor type F	ENSG00000142949	-13.06323917
19	FAM126A	family with sequence similarity 126 member A	ENSG00000122591	-12.91375062
20	TMEM250	transmembrane protein 250	ENSG00000238227	-12.52432039
21	ZNF286A	zinc finger protein 286A	ENSG00000187607	-12.41010853
22	KAT6B	lysine acetyltransferase 6B	ENSG00000156650	-12.04427957
23	TMEM106C	transmembrane protein 106C	ENSG00000134291	-12.00001146
24	ME2	malic enzyme 2	ENSG00000082212	-11.96318188
25	NRN1L	neuritin 1 like	ENSG00000188038	-11.8232807
26	CLN5	CLN5, intracellular trafficking protein	ENSG00000102805	-11.78565591
27	MSL3	MSL complex subunit 3	ENSG00000005302	-11.58713289
28	ZNF550	zinc finger protein 550	ENSG00000251369	-11.5696066
29	ATP6V0E2	ATPase H+ transporting V0 subunit e2	ENSG00000171130	-11.33562392
30	MTHFR	methylenetetrahydrofolate reductase	ENSG00000177000	-11.08995858
31	PNPLA8	patatin like phospholipase domain containing 8	ENSG00000135241	-11.07667048
32	NFXL1	nuclear transcription factor, X-box binding like 1	ENSG00000170448	-11.03801006
33	BRCC3	BRCA1/BRCA2-containing complex subunit 3	ENSG00000185515	-11.03127671
34	CDKN3	cyclin dependent kinase inhibitor 3	ENSG00000100526	-10.92901487
35	NAP1L4	nucleosome assembly protein 1 like 4	ENSG00000205531	-10.86652407
36	POU2F1	POU class 2 homeobox 1	ENSG00000143190	-10.82534134
37	ELMOD3	ELMO domain containing 3	ENSG00000115459	-10.76727553
38	KDSR	3-ketodihydrosphingosine reductase	ENSG00000119537	-10.67483482
39	GIPC3	GIPC PDZ domain containing family member 3	ENSG00000179855	-10.46579233
40	ARV1	ARV1 homolog, fatty acid homeostasis modulator	ENSG00000173409	-10.37292286
41	MSL3	MSL complex subunit 3	ENSG00000005302	-10.25904131
42	TBX15	T-box 15	ENSG00000092607	-10.24942726
43	CD59	CD59 molecule	ENSG00000085063	-10.24286106
44	BTBD3	BTB domain containing 3	ENSG00000132640	-10.21667177
45	PPT1	palmitoyl-protein thioesterase 1	ENSG00000131238	-9.892326871
46	ZNF329	zinc finger protein 329	ENSG00000181894	-9.824692444
47	RNASEH2B	ribonuclease H2 subunit B	ENSG00000136104	-9.819292078
48	GLI2	GLI family zinc finger 2	ENSG00000074047	-9.794869388
49	UBB	ubiquitin B	ENSG00000170315	-9.642763375
50	TBCB	tubulin folding cofactor B	ENSG00000105254	-9.629653352
51	NFATC4	nuclear factor of activated T cells 4	ENSG00000100968	-9.626900238
52	TSPAN9	tetraspanin 9	ENSG00000011105	-9.58185776
53	PUS3	pseudouridine synthase 3	ENSG00000110060	-9.492705899
54	PPP2R3C	protein phosphatase 2 regulatory subunit B''gamma	ENSG00000092020	-9.476290088
55	MRPL19	mitochondrial ribosomal protein L19	ENSG00000115364	-9.447872734
56	NEK10	NIMA related kinase 10	ENSG00000163491	-9.358465372
57	MET	MET proto-oncogene, receptor tyrosine kinase	ENSG00000105976	-9.358283426
58	GNB3	G protein subunit beta 3	ENSG00000111664	-9.256861476
59	NAA60	N(alpha)-acetyltransferase 60, NatF catalytic subunit	ENSG00000122390	-9.240752557
60	HSD17B4	hydroxysteroid 17-beta dehydrogenase 4	ENSG00000133835	-9.230953557
61	DGLUCY	D-glutamate cyclase	ENSG00000133943	-9.222987254
62	CERS6	ceramide synthase 6	ENSG00000172292	-9.188475206
63	CEP162	centrosomal protein 162	ENSG00000135315	-9.184725749
64	GIGYF2	GRB10 interacting GYF protein 2	ENSG00000204120	-9.178548351
65	EIPR1	EARP complex and GARP complex interacting protein 1	ENSG00000032389	-9.004027873
66	GFOD2	glucose-fructose oxidoreductase domain containing 2	ENSG00000141098	-8.966244683
67	CREM	cAMP responsive element modulator	ENSG00000095794	-8.957521663
68	SETDB1	SET domain bifurcated 1	ENSG00000143379	-8.921201142
69	LANCL3	LanC like 3	ENSG00000147036	-8.889128846
70	IL17RC	interleukin 17 receptor C	ENSG00000163702	-8.835096382
71	HIST2H2BF	histone cluster 2 H2B family member f	ENSG00000203814	-8.757173537
72	ZMYM3	zinc finger MYM-type containing 3	ENSG00000147130	-8.603368644
73	PRPSAP1	phosphoribosyl pyrophosphate synthetase associated protein 1	ENSG00000161542	-8.596830377
74	GNE	glucosamine (UDP-N-acetyl)-2-epimerase/N-acetylmannosamine kinase	ENSG00000159921	-8.577657071
75	SLC12A8	solute carrier family 12 member 8	ENSG00000221955	-8.563990542
76	TRIM45	tripartite motif containing 45	ENSG00000134253	-8.556738524
77	INTS14	integrator complex subunit 14	ENSG00000138614	-8.542506039
78	KIAA0753	KIAA0753	ENSG00000198920	-8.526403204
79	TTPAL	alpha tocopherol transfer protein like	ENSG00000124120	-8.526401593
80	ANXA2	annexin A2	ENSG00000182718	-8.504128546
