# Supplementary material for: Gene expression profile analysis of gallic acid-induced cell death process
Source: Sci Rep. 2021 Aug 18;11:16743. doi: 10.1038/s41598-021-96174-1 (PMC8373985; doi:10.1038/s41598-021-96174-1)
Supplement: Supplementary file 2 — Supplementary Information 2. [file 41598_2021_96174_MOESM2_ESM.pdf]

Supplementary Information for Video

Gene Expression Profile Analysis of Gallic Acid-induced Cell Death Process

Ho Man TANG, and Peter Chi Keung CHEUNG [*]

School of Life Sciences, The Chinese University of Hong Kong, Shatin, New Territories, Hong Kong, China

*Co-corresponding author
Peter Chi Keung CHEUNG, Ph.D.
EG09, Science Centre East Block,
Chinese University of Hong Kong,
Shatin, Hong Kong S.A.R.
Tel: (852) 3943 6144
Fax : (410) 2603 5646
E-mail: petercheung@cuhk.edu.hk

Supplementary Video 1

Supplementary Video Legend

Supplementary Video 1. Cell death induced by gallic acid.

Time-lapse confocal microscopy of cytochrome *c*-GFP expressing HeLa cells treated with 50 μg/mL of gallic acid. Merged images of DIC microscopy, cytochrome *c*-GFP (green), nucleus (blue), NucView 530 Caspase-3 substrate for detecting activated caspase activity (red), and IncuCyte Cytotox red reagent for detecting plasma membrane-permeabilization (pink).
